# Nanomaterials: leading immunogenic cell death-based cancer therapies

**DOI:** 10.3389/fimmu.2024.1447817

**Published:** 2024-08-09

**Authors:** Changyu Ma, Zhe Cheng, Haotian Tan, Yihan Wang, Shuzhan Sun, Mingxiao Zhang, Jianfeng Wang

**Affiliations:** ^1^ Department of Urology, China-Japan Friendship Hospital, Beijing, China; ^2^ Graduate School of Peking Union Medical College, Peking Union Medical College, Beijing, China; ^3^ Department of Forensic Medicine, Harbin Medical University, Harbin, China; ^4^ China-Japan Friendship Clinical College, Peking University Health Science Center, Beijing, China

**Keywords:** ICD, nanomaterials, DAMPs, TME, immunotherapy

## Abstract

The field of oncology has transformed in recent years, with treatments shifting from traditional surgical resection and radiation therapy to more diverse and customized approaches, one of which is immunotherapy. ICD (immunogenic cell death) belongs to a class of regulatory cell death modalities that reactivate the immune response by facilitating the interaction between apoptotic cells and immune cells and releasing specific signaling molecules, and DAMPs (damage-associated molecular patterns). The inducers of ICD can elevate the expression of specific proteins to optimize the TME (tumor microenvironment). The use of nanotechnology has shown its unique potential. Nanomaterials, due to their tunability, targeting, and biocompatibility, have become powerful tools for drug delivery, immunomodulators, etc., and have shown significant efficacy in clinical trials. In particular, these nanomaterials can effectively activate the ICD, trigger a potent anti-tumor immune response, and maintain long-term tumor suppression. Different types of nanomaterials, such as biological cell membrane-modified nanoparticles, self-assembled nanostructures, metallic nanoparticles, mesoporous materials, and hydrogels, play their respective roles in ICD induction due to their unique structures and mechanisms of action. Therefore, this review will explore the latest advances in the application of these common nanomaterials in tumor ICD induction and discuss how they can provide new strategies and tools for cancer therapy. By gaining a deeper understanding of the mechanism of action of these nanomaterials, researchers can develop more precise and effective therapeutic approaches to improve the prognosis and quality of life of cancer patients. Moreover, these strategies hold the promise to overcome resistance to conventional therapies, minimize side effects, and lead to more personalized treatment regimens, ultimately benefiting cancer treatment.

## 1 Introduction

Oncology faces the dual challenge of high morbidity and mortality (1), which has become a major challenge in global public health (2, 3). With the aging trend of the population, traditional treatments often struggle to bring lasting relief to patients, which not only increases the socio-economic burden but also has a serious impact on patients’ quality of life. Immunotherapy has shown great therapeutic potential by activating the body’s innate and adaptive immune responses to recognize and remove tumor cells. However, the interaction between tumors and the immune system is far from a simple antagonistic relationship; it also involves a complex process of tumor growth, metastasis, invasion, and recurrence, and may lead to resistance to existing immunotherapeutic drugs (4–8). In addition, the low immunogenicity of tumors poses a challenge to the general activation of immune responses. Thus, a growing body of research suggests that single-agent therapy may not be able to effectively address the complexity of tumors and more precise and integrated treatment strategies are needed.

At the beginning of the 21st century, Professor Guido Kroemer’s research led to the birth of the concept of ICD (immunogenic cell death) when he discovered that cells were able to retain their immunogenicity during tumor cell death induced by the anthracycline chemotherapeutic drug DOX (doxorubicin) (9–11). ICD is a form of regulated cell death that is particularly interesting due to its ability to convert dying tumor cells into a vaccine that stimulates an antitumor immune response. This breakthrough in an interdisciplinary field is expected to solve the technical challenges in immunotherapy. A critical aspect of ICD involves the exposure of calreticulin (CRT) on the cell surface, the release of ATP, and the secretion of HMGB1 (High Mobility Group Box 1), all of which act as ‘danger signals’ or DAMPs (damage-associated molecular patterns). These DAMPs then interact with dendritic cells and other components of the immune system to trigger a robust adaptive immune response. When ICD is induced under stressful conditions, dead tumor cells not only display new antigenic determinants but also release DAMPs (damage-associated molecular patterns), which activate the adaptive immune system. Known ICD inducers include certain chemotherapeutic agents, physical therapies, and pathogens (12–14). However, tumor cells have evolved mechanisms to evade ICD. The therapeutic efficacy of the existing inducers is limited due to their limited variety, poor bioavailability, and poor targeting (15–17).

Therefore, the development of novel and efficient ICD inducers is crucial for tumor therapy. Nanomaterials, with their enhanced permeability, retention effects, and ability to be tailored for specific therapeutic needs, present a promising solution. These materials, such as liposomes, micelles, metallic nanoparticles, and hydrogels, can act as ICD inducers and drug carriers, directly or indirectly inducing immune responses. By leveraging the unique properties of nanomaterials, it is possible to enhance the immunogenicity of tumor cells and improve therapeutic efficacy (18–20).

**Scheme 1:**
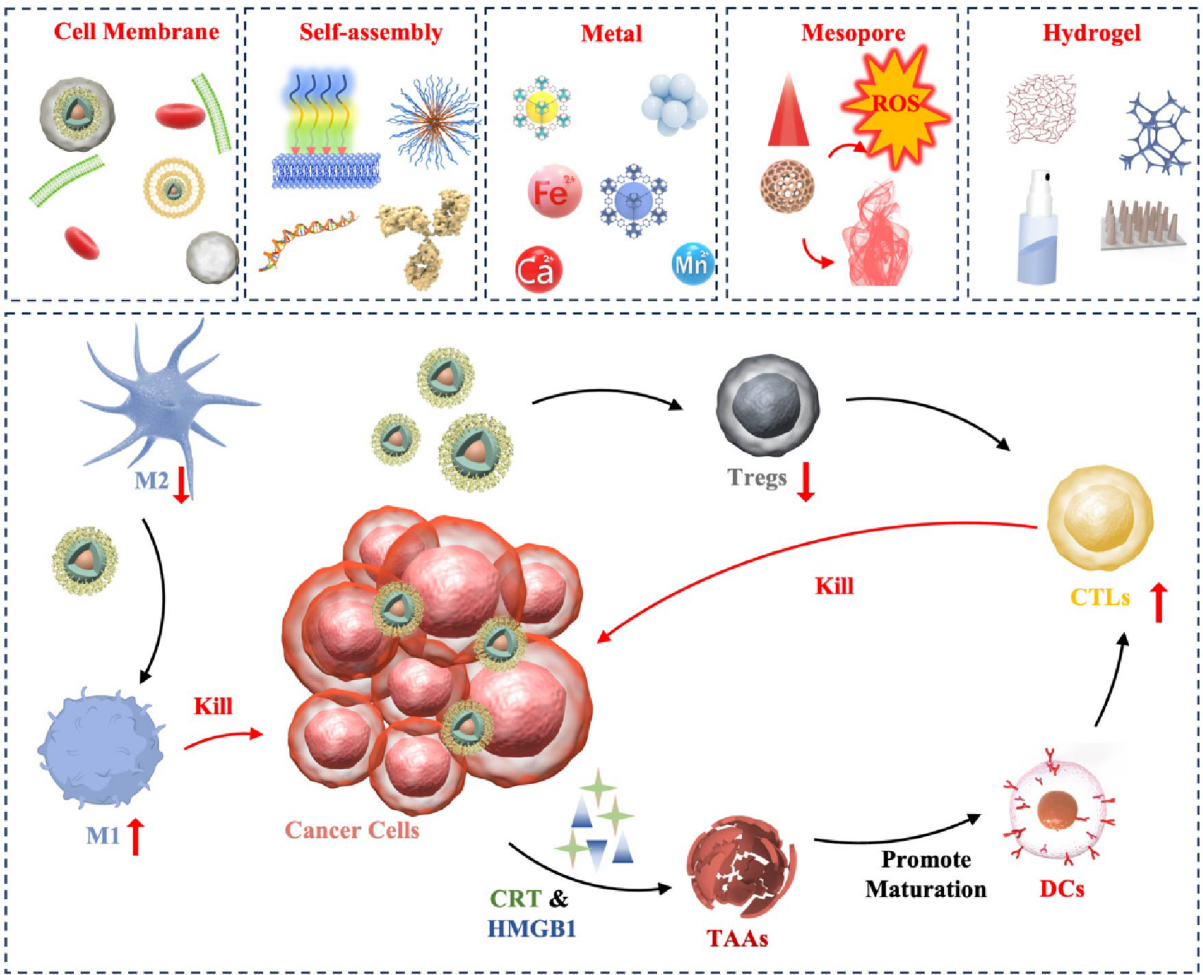
Pattern diagram of the role of nanomaterials in inducing ICD in tumors. These nanomaterials are categorized as cell membrane modified, self-assembly based, metallic, mesoporous, and hydrogel. These nanomaterials exert their anti-tumor effects through ICD in the following major ways: (1) Antigen release: when tumor cells die through ICD, they release TAAs to be captured by DCs, for example. (2) DCs activation: the release of TAAs, as well as HMGB1, etc., promotes the maturation and activation of DCs, allowing them to process and present antigens more efficiently. (3) T-cell response: Activated dendritic cells migrate to lymph nodes and present tumor antigens to T cells, inducing T-cell activation and proliferation. These T cells can then recognize and attack tumor cells carrying the same antigen in the body.

This paper reviews recent advancements in the application of various nanomaterials for ICD induction, focusing on biological cell membrane modification, self-assembled nanostructures, metallic nanoparticles, mesoporous materials, and hydrogels (**Scheme 1**). By combining the potent immune-stimulating effects of ICD with the precision targeting capabilities of nanomaterials, our goal is to develop effective anti-tumor immunotherapy strategies by combining multiple mechanisms and modes of action of conventional therapeutics.

## 2 Cell membrane-modified nanomaterials

Nanomaterials provide a new direction for tumor immunotherapy carriers, which not only enhance the solubility and bioavailability of drugs but also enhance their efficacy by prolonging the half-life of drugs in blood circulation. However, conventional nanocarriers have limitations in terms of stability, targeting, and immune escape. The cell membrane, a complex structure of proteins embedded in a fluid lipid bilayer, not only ensures selective permeability but also mediates communication between cells and between cells and the external environment (21–23). It is responsible for important biological functions such as the transportation of substances, the transmembrane transmission of signals, and energy conversion. Nanomaterials based on cell membranes can effectively circumvent the recognition and removal by the immune system by mimicking the surface properties of natural cells (24, 25). These nanostructures can comfortably navigate through the complex environment of living organisms and achieve precise localization of therapeutic targets.

In addition, due to the diverse and differentiated functional properties of cell membranes, we can tailor the nanocarriers to specific therapeutic needs by selecting different cell membrane types. This strategy not only improves the targeting of nanomaterials but also can effectively stimulate ICD. This chapter provides an in-depth analysis of the application of biomaterials possessing structural features of cell membranes, such as tumor cell membranes, erythrocyte membranes, platelet membranes, and liposomes, in the surface modification of nanocarriers, as well as the effects and possibilities of these modifications to stimulate ICD. We expect that these leading cell membrane modification technologies will significantly enhance the functionality of nanocarriers and open up innovative avenues for the development of tumor immunotherapy.

### 2.1 Tumor cell membrane-modified nanomaterials

Studies have revealed that vesicles originating from cancer cell membranes can significantly enhance the effectiveness of tumor therapy by their homologous targeting ability and inherent immune evasion mechanism. While nanomaterials often exhibit well anti-tumor activity in the *in vitro* experimental setting, in the *in vivo* setting, they often fail to achieve the desired therapeutic effects due to their insufficient targeting and stability. Researchers have found that the use of tumor cell membranes to modify nanoparticles can enhance drug delivery efficiency while specifically transporting drugs to tumor cells, which not only serves as a platform for drug delivery but also helps to stimulate a strong anti-tumor immune response *in vivo* by providing tumor-specific antigens (26–28).

Tumor cell membrane drug delivery systems are often used as adjuvants in combination with other immunotherapeutic strategies to achieve more significant antitumor efficacy due to their superior targeted delivery ability and prevention of immune escape. The advantages of PDT (photodynamic therapy) as a photosensitizer- and laser-activated tumor treatment rely on minimal systemic toxicities under local irradiation conditions, as well as the low drug resistance demonstrated during repeated treatments. However, PDT has not performed sufficiently in stimulating anti-tumor immune responses, resulting in its efficacy in completely eradicating tumors and their metastases have not yet reached an ideal state (29–31). Li et al. (32) designed MON nanoparticles using OVA (antigenic ovalbumin) to carry photosensitizer Ce6 to form ON and then coating ON with B16-OVA cancer cell membranes. The uptake of MON by homozygous cancer cells was much higher than that of the free Ce6 and ON groups, suggesting a homologous targeting effect. *In vitro* antigen cross-presentation assay analysis yielded that MON is crucial in promoting BMDC maturation, triggering ICD. MON is a stimulant that promotes DC maturation under laser irradiation. At the same time, MON upregulates CCR7, CD80, and MHC-II under laser irradiation and stimulates DC to express more IL-12, TNF-α, and IL-6. In the B16-OVA tumor-bearing mouse model, MON exhibited potent tumor suppressive effects. The team verified the immune resistance by melanoma cells attacking MON in mice treated with light, and the results showed that the ratio of memory cells in CD4^+^ T to CD8^+^ T cells in the spleen of mice was significantly increased. The final results suggest that the design and application of MON are promising for triggering ICD and complementing PDT for tumor killing. Similarly, Zhao’s team (33) used 4T1 cell membranes encapsulated with CMO nano-enzymes with multi-enzyme mimetic properties, the photosensitizer R848, and a TLR7/8 agonist. The nanomaterials NIR-II light produced a photothermal effect in which the nanoenzymes promoted the production of ROS (reactive oxygen species), and they synergized with the TLR7/8 agonist to induce immunogenic cell death in tumor cells and maturation of DCs, which enhanced the immune response.

**Figure 1:**
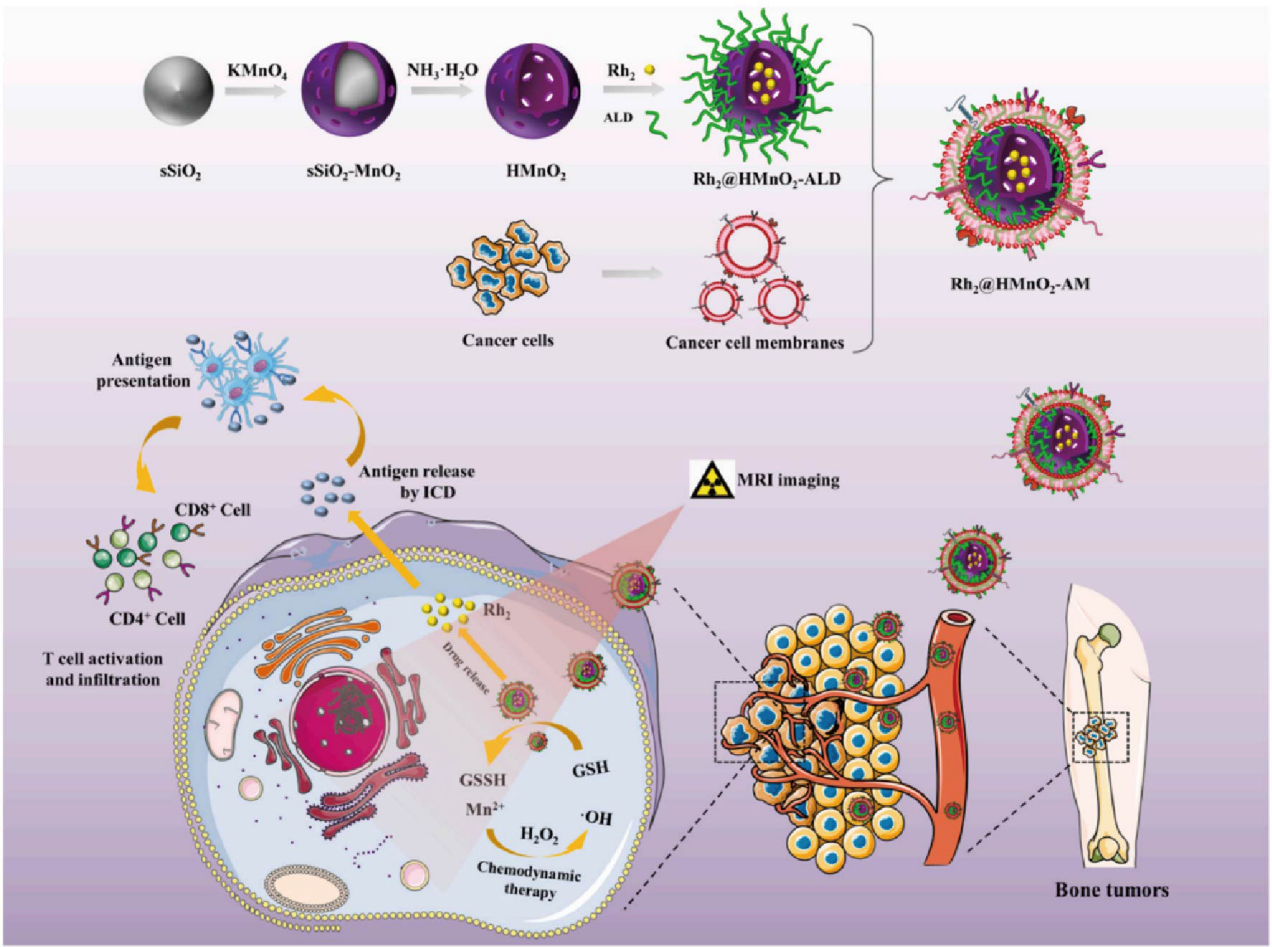
Rh_2_@HMnO_2_-AM synthetic model diagram and mechanism of MRI-guided chemodynamic therapy and ICD synergistic treatment of osteosarcoma (34). Copyright^©^ 2022, copyright Fu et al.

To achieve efficient anti-tumor purposes and solve the problem of poor targeting and inefficiency of tumor therapy, the development of a TME (tumor microenvironment)-triggered drug delivery system provides new ideas. It has been proposed that modulating the TME in tumors can significantly improve the efficiency of cancer therapy. Based on the unique TME responsiveness of MnO_2_ nanoparticles, He’s a team (34) designed a Rh_2_@HMnO_2_-AM nanosystem formed by K7M2 cell membranes wrapped with hollow MnO_2_ and then loaded with ginsenoside Rh_2_. The system is pH-sensitive and GSH-sensitive, with mild cytotoxicity and good cytocompatibility. *In vitro* experiments, flow cytometry measurement of DC, and characterization of markers showed that Rh_2_@HMnO_2_-AM induced ICD in tumor cells by massively expressing HMGB1 with upregulation of BAX, BCL-2, and Caspase 3 levels. Rh_2_@HMnO_2_-AM nanoparticles showed higher anticancer efficiency (**Figure 1**). The tumor immunotherapeutic mechanism of Rh_2_@HMnO_2_-AM-induced Tregs reduction in a mouse *in situ* K7M2 tumor model led to more cancer cell death after using Rh_2_@HMnO_2_-AM nanoparticles, and the survival time of mice exceeded far longer than the other groups. These results emphasize that Rh_2_@HMnO_2_-AM is an excellent TME-activated nanoparticle with immunotherapeutic and anti-metastatic potential in cancer treatment. Overall, this study successfully combines immunotherapy and CDT (chemodynamic therapy) to develop a new modulation strategy that promotes new developments in tumor and MnO_2_ materials.

Although developing new chemotherapeutic drug targets provides new perspectives for tumor treatment, the drawbacks of low stability and bioavailability and poor targeting make the drugs less effective in killing tumors. To address the interference of physiological barriers to micelle accumulation in tumors, modification of nanomaterials using tumor cell membranes offers a new idea for tumor vaccine development (35–37). Shen’s team (38) designed CM camouflage with redox reactivity and 1G_3_ Cu/Toy loaded polymeric micelles GCT@CM NPs. The CM coating imparts good anti-fouling properties to the micelles properties to resist protein adsorption and facilitate prolonged *in vivo* blood circulation of NPs. At lower GSH concentrations, NPs can still dissociate responsively, solving the problem of low nanoparticle stability. *In vitro* experiments showed that GCT NPs exhibited significant GSH depletion effects and generated more ROS, which aggravated oxidative stress. Signal intensity was significantly enhanced. Intravenous injection of GCT@CM NPs could lead to tumor cell apoptosis by inducing oxidative stress, amplifying endoplasmic reticulum stress, and causing mitochondrial dysfunction under the action of CM towards the tumor site. Meanwhile, the team combined GCT@CM NPs with anti-PD-L1-mediated ICB (immune checkpoint blockade) treatment could significantly inhibit tumor growth and obtain optimal therapeutic effects. In summary, the innovative application of CM-modified drugs in the study enabled the drugs to reach their targets smoothly and efficiently, and it is believed that nanomaterials such as GCT@CM NPs will shine in clinical treatment.

Functionalized nano-delivery systems with specific drug transport have excellent application prospects. The application of tumor cell membranes in bionic drug delivery systems has excellent advantages. Tumor cell membranes can retain antigens on the cell membrane to stimulate immune responses and maintain isotype adhesion for targeted delivery. Huang’s team (39) designed cancer cell membrane-encapsulated TPP (triphenylphosphine) -modified nano-metal organic framework structures. This nanomaterial-induced ICD promotes DC maturation. It also activates immune memory and helps to establish durable anti-tumor immunity. Combined with anti-CTLA-4, it demonstrates a more potent therapeutic ability. Similarly, Luo et al. (40) obtained mEHGZ by ZIF-8 nanoparticles encapsulating epirubicin, glucose oxidase, and heme chloride and wrapped with CRT overexpressing 4T1 cell membranes. Interestingly, mEHGZ strongly induced the transport of CRT from the endoplasmic reticulum to the cell membrane, a type of cell that can induce DC cell uptake and activation of the immune response by signaling proteins. The results showed that the intervention of mEHGZ nanoparticles led to significant retardation of tumor growth, demonstrating a potent antitumor effect. Overall, these cancer cells’ membrane-modified nanoproducts based on triggering ICD leading to apoptotic tumor necrosis exhibit unparalleled superiority of raw materials and are worth investing time and effort to study in depth.

Although DOX can induce the ICD of tumor cells, its clinical application is limited by both its severe cardiotoxicity and the development of chemotherapeutic resistance to it by tumor cells. Of particular concern is the role of ROS overproduction in DOX-induced cardiotoxicity, which not only leads to apoptosis of cardiomyocytes but also activates a variety of cardiac fibrosis-promoting factors, among which TGF-β1 is particularly important. TGF-β1 not only further induces apoptosis in cardiomyocytes, but is also a key cytokine that drives the formation of CAFs (cancer associated fibroblasts), playing a role in promoting tumor development in the TME (41–43). Therefore, addressing the cardiotoxicity of DOX, as well as overcoming its chemotherapeutic resistance, is of great significance for improving the therapeutic efficacy of DOX and the quality of patient survival. Sun et al. (44) utilized tumor cell membrane-encapsulated DOX and siTGF-β1 (TGF-β1 siRNA) to induce tumor cells to undergo immunogenic cell death, while siTGF-β1 was able to modulate the TME by precisely and efficiently silencing TGF-β1 expression and blocking the TGF-β1 signaling pathway, thereby enhancing the antitumor effect of DOX. This synergistic effect has also been applied to ferroptosis-related nanomaterials. Wang et al. (45) constructed a complex consisting of Salmonella typhimurium VNP (*Salmonella typhimurium*, VNP20009), Fe_3_O_4_ and tumor cell membrane. Tumor cell membrane-modified Fe_3_O_4_ nanoparticles effectively inhibited tumor growth through the potentiating effect of ferroptosis inducer sulfasalazine and generated an immune response through ICD. In addition, the settlement of VNP induced an adaptive immune response and further promoted ferroptosis.

Tumor cell membrane-encapsulated nanomaterials offer unique advantages in inducing ICD in tumors, including homologous targeting, reversal of immune escape, good biocompatibility, and enhanced immune activation. The aforementioned advantages make it an exciting area of research with the potential to change our perception and practice of tumor therapy.

### 2.2 Erythrocyte and platelet membrane-modified nanomaterials

Erythrocytes are a biconcave morphology with the property of changing shape in response to changes in surrounding osmotic pressure, which provides a basis for binding erythrocytes to nanomedicines. Erythrocytes provide 120 days of blood circulation, and erythrocytes increase the accumulation of nano- and microparticles when narrow microvessels are constricted (46, 47). In addition, erythrocytes are typical of long-term circulating delivery vehicles *in vivo* due to their remarkable biocompatibility and non-immunogenic properties. Notably, senescent erythrocytes can be cleared by macrophages and dendritic cells. Similarly, platelet membranes can also produce the same functions as erythrocyte membranes (48–50).

In summary, the advantages of erythrocyte-loaded nanoparticles as carriers are as follows: (1) good biocompatibility and biodegradability; (2) evasion of immune response and prolonged circulation time; (3) a lifespan of 120 days; erythrocyte-modified nano drugs as an “invisible cloak” for immune escape and a “guide” for tumor targeting have been widely used. “erythrocyte-modified nano drugs have been widely used in tumor therapy and have achieved significant results (51, 52). Studies have shown that tumor immunotherapy with immunogenicity, which refers to the ability to induce an immune response in the host, can play an active role in treatment. Reversing immunodeficient “cold tumors” to immunogenic “hot tumors” is challenging to achieving maximum therapeutic benefit (53, 54).

Currently, multifunctional nano bullets with erythrocyte membrane (RBCm) cloaking properties are widely investigated, and Tian et al. (55) developed a nanostructure with a heat-sensitive SNO donor side chain copolymer PAAV-SNO as the core and erythrocyte membrane as the shell. The stealthy properties and extended circulation properties of the erythrocyte membrane are utilized. Meanwhile, _Δ_T of RBCm/PAAV-SNO NPs was approximately constant after three consecutive cycles of laser and non-laser irradiation, indicating good photothermal stability. In *in vitro* experiments, RBCm/PAAV-SNO NPs significantly reduced the upregulation of PD-L1 expression in hypoxia-induced 4T1 cells under NIR-II laser irradiation, exerting an immune checkpoint inhibitory effect. In addition to this, the levels of CRT and HMGB1 were significantly upregulated, Tregs were reduced, the expression of CD8^+^ T cells was increased, and the release of INF-γ and granzyme B was increased, indicating the promoting effect of RBCm/PAAV-SNO NPs on ICD. Within the tumor model, it is exciting that the modification of RBCm significantly prolonged the blood circulation of NPs and achieved good passive targeting ability. All the advantages prove that this nano-bullet is convincing in eliminating cancer cells through the photothermal effect. If clinically translated, it will bring blessings to countless cancer patients.

Nanomedicines have non-negligible limitations in tumor treatment due to their defects of low targeting and poor stability, and some studies have shown that the application of blood cells provides new directions for the improvement of new nanomaterials. The application of red blood cells, with their inherent biocompatibility, flexible surface, and large cavities, provides a safe passage to tumor sites for the entire nanomaterial. Min’s research team (56) designed a new nanomaterial, RBC BSA, constructed first with tumor antigens (cAg) induced by chemotherapeutic agents causing ICD, followed by red blood cell modification. The scheme aims to exploit the properties of red blood cells to achieve specific delivery of nano drugs. *In vitro* experiments showed that RBC BSA significantly upregulated the expression levels of CD80 and CD86 and promoted the maturation of approximately 60% of immature BMDCs. Combined treatment with RBC vaccine and PD-1 blocker was employed within a CT-26 homozygous mouse model, resulting in surface combination treatment increased the levels of CD4^+^ and CD8^+^ T cells in tumors and significantly increasing IFN-γ expression within CD8^+^ T cells. Tumor re-invigoration experiments showed that combination treatment with the RBC vaccine and αPD-1 induced the expansion of memory T cells, thus helping to establish long-term immunity and prevent tumor recurrence. Notably, with the help of RBCs, the vaccine is more likely to exert more substantial efficacy and eventually invest a non-negligible contribution to the field of tumor therapy.

PDT has been widely used in tumor treatment due to its extremely low invasiveness and precise spatiotemporal selectivity. ROS is considered an effective option to eradicate cancer cells, but tumor hypoxia limits the outcome of PDT and the progression of ICD. To address this issue, Teng’s team (57) used PM (platelet membranes) as nanocarriers to encapsulate Met (metformin) and IR780 to obtain PM-IR780-Met NPs. As blood components, PM can serve as transport carriers for IR780 and Met to evade immunosurveillance and physical clearance to ensure the stability of the nanomaterial transport process. *In vitro* experiments, PM-IR780-Met NPs showed increased levels of ROS release and significantly reduced ATP content, alleviating hypoxia from both perspectives and thus exerting a more powerful PDT effect. Meanwhile, PM-IR780-Met NPs triggered higher levels of CRT and HMGB1, which facilitated the stimulation of DC maturation and T cell activation, suggesting that PM-IR780-Met NPs have a promising performance for the induction of ICD. PM-IR780-Met NPs induced systemic and anti-tumor solid immune responses, showing significant ablation of tumor volume. NPs showed extensive necrosis and apoptosis in tumor tissues when applied synergistically with laser, and no cancer metastasis occurred in lung tissues, demonstrating powerful tumor therapeutic effects. At the same time, minor changes were found in the body weight and serum biochemistry of the mice tested. This suggests the excellent biosafety of PM-IR780Met NPs. Overall, nanomaterials play a good role in tumor growth and metastasis with full clinical translational potential, which is worth to be learned from more studies.

The application of bionanomaterials provides a solution to the problem of targeting and stability in drug delivery. Considering the immune function of erythrocytes and platelets, using them as carriers for antigen delivery is not only effective in inducing immune responses but also reduces the toxic side effects of the organism. Guo et al. (58) designed a tumor-targeting-enhancing nanoformulation encapsulated by erythrocyte membranes using PLGA to co-encapsulate PLB, DIH, and NH_4_HCO_3_. The study showed that the nanoformulation could The nano preparations were shown to upregulate immunostimulatory cells in tumors, such as DCs and NK cells, and downregulate immunosuppressive cells in tumors, such as Tregs and MDSCs, producing a reversal of immunosuppressive TME and enhancing systemic antitumor immunity against tumor recurrence and metastasis. In addition, Wang’s team (59) synthesized a bionic decoy, Lipo-Ce6/TPZ@MH, which uses erythrocyte-PLTs MH (hybrid membrane) to camouflage Ce6 with TPZ and exhibits potent cancer accumulation and retention capabilities. In *in vivo* experiments, Ce6/TPZ@MH exerted powerful therapeutic effects of substantial tumor accumulation and prolonged blood retention under US treatment, leading to several 100% tumor growth inhibition rates. In addition, the system possesses low cytotoxicity and good biosafety, hence in clinical treatment. Overall, this nanoformulation’s therapeutic efficacy and safety are positive, and this cascade treatment strategy is worthy of being studied and applied in the follow-up work.

Erythrocyte and platelet membrane-modified nanomaterials have the following unique advantages in inducing ICD in tumor cells: (1). Long circulation time: Erythrocyte and platelet membranes have natural “camouflage” properties that can avoid recognition by the immune system, thus prolonging the lifespan of the nanomaterials in the blood circulation, which helps to improve the accumulation of the drug at the tumor site and the therapeutic effect. (2) Biocompatibility: Since red blood cells and platelet membranes are extracted from autologous or allogeneic biological materials, they are highly biocompatible, reducing immune rejection and toxic effects. (3). Natural targeting: platelets naturally tend to aggregate in injured blood vessels and TME, so platelet membrane-modified nanomaterials may have natural tumor-targeting properties. Erythrocyte membranes may also interact with cells in the TME via specific cell surface proteins. (4). Immune escape: erythrocyte and platelet membrane-modified nanomaterials can mimic the surface characteristics of natural cells, which helps them to avoid being recognized and cleared by immune cells, thus reducing immune clearance. (5). Reduced risk of blood coagulation: due to the natural origin of red blood cells and platelet membranes, they reduce the risk of coagulation when interacting with blood components, which is particularly important for intravenous nanomedicine delivery systems. These advantages make erythrocyte and platelet membrane-modified nanomaterials potentially promising in the field of tumor therapy, where they can be used as an effective strategy to enhance ICD and promote tumor immunotherapy.

### 2.3 Liposomes-modified nanomaterials

Liposomes are new drug carriers extensively investigated in oncology therapy. Liposomes are closed vesicles with an internal aqueous phase formed by lipid bilayers, which have significantly contributed to the solution of drug carrier biocompatibility, biodegradability, cytotoxicity, immunogenicity, etc. The main types of cysts are small single vesicles, multilamellar vesicles, and large monolamellar vesicles. Conventional liposomes are mainly passively targeted to tumor tissues with the help of the EPR effect but still have the disadvantages of low targeting efficiency and poor specificity and are prone to off-target effects in tumor tissues (60–62). Therefore, designing new liposomes with an active targeting effect has excellent application prospects.

Liposomes are naturally targeted due to their physiological properties as macrophages easily phagocytose them as foreign bodies and accumulate in organs or tissues such as the brain, liver, and spleen. DOX, a first-line drug for breast cancer treatment, is an anthracycline that can serve as an effective ICD inducer. Preliminary experimental data from Nel’s team (63) showed that DOX could effectively induce ICD responses by internal liposome encapsulation in an *in situ* animal model. On this basis, the team constructed DOX/IND liposomes using self-assembly of the phospholipid-coupled prodrug IND (indomethacin), which inhibits the IDO-1 pathway, followed by remote loading of DOX. Because of the fluorescent drug property of DOX, detection of fluorescence intensity in mouse tumor models could qualitatively determine the distribution of the drug in tumors. The results showed a 10-fold increase in fluorescence intensity in mice injected with DOX/IND liposomes or DOX NP. Similarly, encapsulated liposomes also acted as a good deliverer of IND. DOX/IND liposomes triggered ICD, which reduced the primary tumor size in mice to 1/3 of the original size *in vivo* experiments. The team combined DOX/IND liposomes with anti-PD-1 antibodies to enhance the efficacy. The results showed a significant reduction in tumor size and complete disappearance of lung tissue metastases in the animals. This study shows that liposome vectors are advantageous in inducing ICD, and synergistic treatment with anti-PD-1 plays an excellent therapeutic effect.

Liposomes are the most successful nanodrug carriers for clinical application with specific passive targeting abilities based on high permeability and EPR effects. The modified liposomes can specifically bind to the target cells and enrich the drug at the tumor, thus improving the therapeutic effect while reducing the drug’s toxic side effects on normal tissues. Based on these studies, Liu’s team (64) constructed bifunctional liposomes aNLG/Oxa(IV)-Lip by self-assembling oxaliplatin and NLG_919_ prodrugs and commercial lipids. Immunofluorescence staining showed significant release of CRT and HMGB1 from CT26 cancer cells after receiving bifunctional liposomes, demonstrating that oxaliplatin and its liposome formulation induced effective ICD. Injection of aNLG/OXA(IV)-Lip showed that tumors grew less than the rest of the groups within 8 days and began to shrink in size after 8 days; similarly, mice survived considerably longer after bifunctional liposome treatment, were cured within 90 days, and no significant recurrence was observed. This suggests that the most effective inhibition of bifunctional liposomal tumor growth was demonstrated. In addition, aNLG/OXA(IV)-Lip showed negligible effect on the body weight of these mice during treatment at the same therapeutic dose, and liposome biocompatibility was ensured. Also, aNLG/Oxa(IV)-Lip induced more potent tumor immunity by promoting DC cell maturation, intratumoral infiltration of CD8^+^ T cells, and secretion of TNF-α and IFN-γ. In conclusion, this study of combining liposomes with chemotherapeutic agents through a simple self-assembly method resulting in strong tumor-killing effects is expected to be further optimized and applied in clinical treatment.

The liposome encapsulation of DOX and oxaliplatin makes them less susceptible to degradation in the *in vivo* environment, enhances drug stability, and improves the efficacy of tumor therapy. However, the extensive utilization of both drugs has led to frequent occurrences of resistance to both drugs in organisms. Therefore, the development of new drugs is necessary for future tumor therapy. Nel et al. (65) focused on MTO (mitoxantrone), a drug not commonly used in solid tumor chemotherapy, by remotely introducing the anthraquinone chemotherapeutic agent MTO into liposomes and generating MTO/IND dual-delivery liposomes with cholesterol-bound IND prodrugs. Previous work found higher CRT expression and HMGB1 release with the same dose of MTO than with DOX and oxaliplatin, suggesting a more potent ICD-inducing effect of MTO. Within the CT26 cancer model, co-delivery of MTO with IND produced much higher and slower decreasing MTO concentrations than free drug at 24 and 48 hours. Intravenous administration of MTO/IND liposomes significantly enhanced the immunotherapeutic response leading to a significant reduction in tumor size, as evidenced by the appearance of ICD markers and cytotoxic cancer cell death mediated by perforin and granzyme B. Notably, the cytotoxic effect involved NK cells, which suggests a different type of ICD response. It also prolonged animal survival beyond the effect of using MTO liposomes alone. Besides CT26 colon cancer, MTO/IND liposomes effectively inhibited tumor growth in breast cancer and kidney cancer models.

It has been found that in addition to the above applications, synergistic treatment of liposomes with the immune checkpoint PD-1 can enhance the efficacy of liposomes. Liu’s team (66) encapsulated liposomes constructed from metformin and amphiphilic oxaliplatin prodrugs to obtain met-oxa(IV)-liposomes, a TME-modulating liposome nanodrug. The physiologically stable liposomes gradually release oxaliplatin and metformin, which trigger ICD killing of cancer cells by upregulating CRT and HMGB1 expression. Isolated fluorescence imaging measurements of tumor accumulation showed that the tumor accumulation of met-oxa(IV)-liposomes and oxa(IV) liposomes were calculated to be 5.96% and 6.98%, which is approximately 5-7 times the accumulation of free oxaliplatin. Liposome treatment may also synergize with anti-PD-1 ICB treatment to produce enhanced tumor treatment and prolong survival time in mice. In addition to acting as a “guardian” of nanomedicines, modified liposomes can also be used to construct photothermal sensors with better performance. And NIR-II biological windows with different absorption of PTT (photothermal therapy) transducers. *In vivo*, NIR-II photothermal therapy resulted in a more uniform release and distribution of DAMPs deep in the tumor. Along with ICD production, NIR-II PTT triggers innate and adaptive immune responses, keeping most mice tumor-growth-free in cancer vaccination experiments. The results show that NIR-II PTT is more effective in penetrating tissues and treating deep tumors and is worthy of application in human therapy.

Liposome modification of nanomaterials provides a highly versatile and customizable platform for inducing ICD and executing tumor immunotherapies, opening up new strategic avenues and potential opportunities for cancer therapies. One notable example of such technological advancements is Vyxeos^®^Liposomal, a 100 nm bilayer liposome nanoparticle specifically designed to carry the combined chemotherapy drugs cytarabine and daunorubicin at a synergistic molar ratio of 5:1. This drug was approved by the United States Food and Drug Administration (FDA) in 2017 for the treatment of acute myeloid leukemia (AML). In a pivotal efficacy study (NCT01696084), Vyxeos^®^Liposomal significantly improved overall survival, reaching 9.6 months (p-value=0.005), compared to 5.9 months in the control group without the drug (67). These findings underscore the profound impact of liposome-based nanotherapies in clinical settings, highlighting their potential to enhance treatment outcomes and provide new hope for patients with challenging diseases such as AML.

The unique advantages of liposome-modified nanomaterials in inducing ICD in tumor cells include (1). Biocompatibility and biodegradability: liposomes are composed of natural or synthetic phospholipids and cholesterol. These properties make them relatively safe for the organism during metabolism and excretion in the body. (2). Mimicking the structure of biological membranes: the bilayer lipid structure of liposomes resembles cell membranes, which facilitates effective fusion and interaction with cell membranes and promotes cellular uptake of drugs. (3). Drug encapsulation ability: liposomes can effectively encapsulate a wide range of drugs, including water-soluble and fat-soluble drugs. They can encapsulate drugs in their aqueous-phase core, lipid bilayer, or surface modifiers, thus providing multiple drug delivery options. (4). Controlled release kinetics: liposomes can be designed to be responsive, e.g., to release drugs under low pH or high enzyme activity conditions in the TME, improving therapeutic selectivity and reducing side effects on normal tissues. (5). Targeted delivery capability: the surface of liposomes can be modified with targeted ligands (e.g., antibodies, proteins, peptides, etc.), enabling them to bind specifically to receptors or antigens on the surface of tumor cells for targeted delivery. Liposome modification of nanomaterials provides a highly versatile and customizable platform for inducing ICD and executing tumor immunotherapies, opening up new strategic avenues and potential opportunities for cancer therapies.

## 3 Self-assembled nanomaterials

The application of bio-nanotechnology in the field of drug delivery has given new impetus to the development of high-end innovative formulations. Self-assembly is the spontaneous arrangement of molecules into ordered structures. Compared with conventional nano drug delivery systems, self-assembled nano drug delivery systems have unique advantages, such as flexible and specific preparation methods, high drug loading capacity and delivery efficiency, the long half-life of *in vivo* circulation, and low toxicity. For example, Zhang et al. (68) developed a detachable core-shell nano protein by self-assembly that not only kills tumors but also has an educational effect on TIME; Yao’s team (69) also designed nanoparticles DAR by self-assembly that enables efficient ferroptosis immunotherapy; Kim’s team (70) designed low toxicity, low immunosuppressive nano-agent CAP NP also by self-assembly. Many other schemes like this allow altered drug properties to enhance therapeutic efficacy utilizing self-assembly, and the following sections will review the application of self-assembly strategies based on proteins and nucleic acids, metals, and polymers in inducing the immunogenic death of tumors.

### 3.1 Nanomaterials based on self-assembly of nucleic acids, peptides and proteins

Nucleic acids, polypeptides, and proteins are the basic structures that make life and are closely connected. Nucleic acids are the essential components of cells and play important roles in various biological processes. Nucleic acid materials have gained significant interest due to their excellent biocompatibility and programmability. Peptides are short-chain molecules composed of amino acids linked by amide bonds and can be used to build proteins by giving them a spatial structure. Both peptides and proteins have the advantage of good modifiability and safety, and their structures determine their biological functions. It can be seen that all three have the basis for clever combination with self-assembly, and delicate design can allow them to spontaneously form different assembly forms to form specific nanostructures for applications in targeted drug delivery, cell imaging, and other fields. Researchers have developed a series of nucleic acid-, peptide- and protein-based therapeutic solutions (71–73).

Since the first successful preparation of tFNA (tetrahedral framework nucleic acids) by Turberfield, a wide variety of tFNA functionalization strategies have been widely explored. Due to their appropriate size and geometry, tFNA is highly efficient for cellular endocytosis and tissue penetration. At the same time, its inherent programmable structure allows researchers to develop tRNA-related materials specifically. Based on the above background, Lin et al. (74) constructed a nanostructure CpG tFNA/DOX formed by self-assembling the tFNA vector with DOX. DLS showed that CpG tFNA/DOX had a size of 39.24 ± 1.76 nm and a stable structure that could prolong the degradation time of drugs *in vivo*. Besides, the membrane permeability of tRNA facilitated more entry of nano drugs into the cells. CpG tFNA/DOX increased CRT levels and enhanced HMGB1 secretion and ATP release, indicating nanoparticles’ positive effect on inducing ICD and anti-tumor immune response in tumor cells. CpG-tFNA/DOX in B16 rheumatoid mice showed in tumor tissue. After treatment of B16 tumor-bearing mice with control and experimental groups, increased CD80 and CD86 expression in the tumor and spleen and increased CD8^+^ T cells could be observed in the CpG tFNA/DOX group after treatment. To further increase the efficacy, the team combined CpG tFNA/DOX with anti-PD-L1 treatment, which showed a further increase in mature DCs and CD8^+^ T cells. Meanwhile, the combination significantly enhanced IFN-γ and TNF-α levels, which could significantly inhibit tumor growth. In summary, this nanoparticle synergizes with αPD-L1 to further induce tumor immunity by triggering ICD to promote apoptosis, and this self-assembled nanomaterial is a promising approach to build a “bridge” between drugs and immunotherapy.

**Figure 2:**
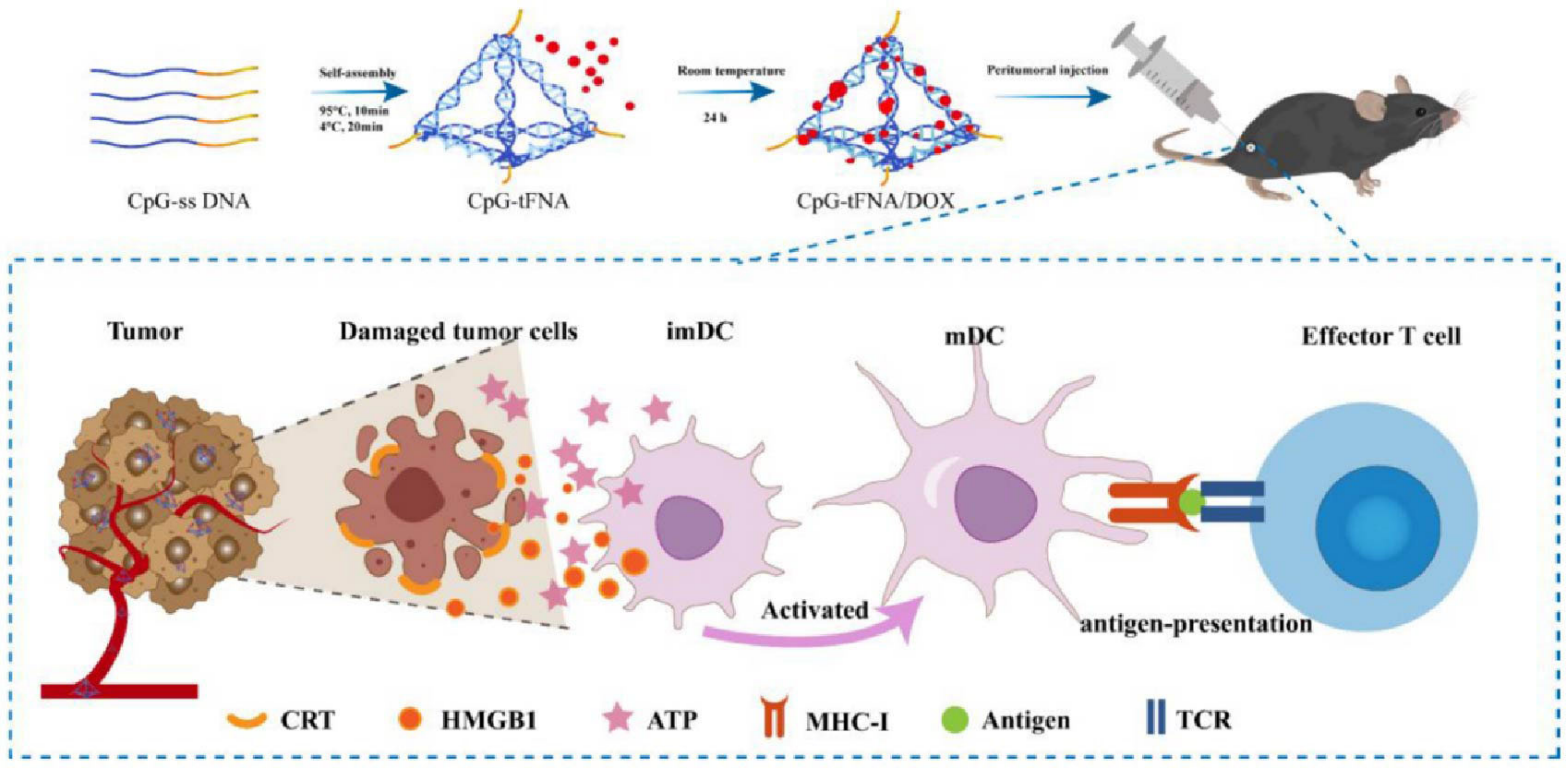
Schematic diagram of CpG-tFNA/DOX preparation and ICD induction in tumor cells (75). Copyright^©^ 2022, copyright Liu et al.

ICB has shown remarkable results in treating many cancers by blocking immunosuppression. Among them, therapies using anti-PD-1 or anti-PD-L1 antibodies are widely used. However, conventional PD-L1 antibodies suffer from poor permeability, low drug delivery efficiency, difficulty in mass production, and toxicity, and the combined application of other therapeutic approaches provides a new solution to these problems. Kim et al. (75) constructed a visible light nanoparticle LT NPs using a self-assembly method.LT NPs combine photosensitizers VPF (vetiporfin), histone B-specific cleavable peptide, and DOX (**Figure 2**). LT NPs exhibited efficient ROS generation efficiency (90%) upon incubation with histone B enzyme, suggesting that drug toxicity at off-target sites was mitigated. In *in vitro* experiments, the results showed that LT NPs were mainly concentrated in the cytoplasm. In contrast, VPF and DOX were concentrated in the cytoplasm and nucleus, respectively, confirming the excellent cleavage and targeting properties of the nanoparticles as expected. In addition, increased CRT expression in LT NPs + light irradiation-treated CT26 cells demonstrated the ability of LT NPs to induce ICD upon photoactivation. The increase in other DAMPs and the maturation of DCs (increased CD45^+^CD3^+^CD8^+^) further flesh out this argument. As expected, the team’s synergistic treatment of LT NPs with anti-PD-L1 inhibited tumor growth and produced a promising anti-tumor immune response through immune memory. In addition, the combination treatment significantly reduced metastatic nodules and lung metastases and significantly prolonged tumor survival in mice compared to other treatments. In short, the combination of LT NPs therapy provides a new strategy for tumor treatment by enhancing both cellular uptake and efficacy compared to anti-PD-L1 alone.

The tumor immune microenvironment leads to the immune evasion of tumors, in which TAMs play an essential role. Therefore, depletion and modification of TAMs can help to re-establish the tumor immune microenvironment and achieve immune normalization. Huang’s team (76) constructed a mannosylated lactoferrin nanoparticulate system Man-LF-NP for the co-delivery of paclitaxel and JQ1 by self-assembling paclitaxel (SHK), and JQ1 encapsulated in lactoferrin nanoparticles. The self-assembly process resulted in a much higher drug loading efficiency and drug encapsulation efficiency of Man-LF-NP than that of the drug alone. CRT exposure on CT26 tumor cells was notably significantly higher, and HMGB1 release was substantially increased after Man-LF-NP treatment. Also, nano-drug-treated tumor cells were more immunogenic than free drugs, inducing up to 83% of mature DCs. Man-LF-NP also played a role in TAM reprogramming. Man-LF NP resulted in the downregulation of M2-related markers and increased expression of STAT1 and TNF-α, which represented a blocking effect on converting TAM1 to TAM2. Treatment with Man-LF NP decreased TGF-β expression, promoted anti-tumor TNF-α production, and enhanced anti-tumor immune effects. Nanoparticles constructed using the self-assembly process exhibited considerable tumor-damaging effects, and therefore, developing more self-assembly-based nanomaterials is necessary for effective anti-tumor therapy.

Looking back at the history of human development, vaccines are an unprecedented milestone in using the human immune system to treat diseases and save lives. Compared with conventional vaccines, nano vaccines have the following advantages: (1) customizability, (2) improved drug stability, (3) function as immune adjuvants, and (4) facilitate lymph node accumulation. However, nano vaccines’ low loading rate and poor permeability require improvement. He’s team (77) designed a redox-reaction-based nanocomponent immunotherapy regimen, R-mPDV/PDV/DOX/siL, which was constructed by self-assembling three glutathione (GSH) polymers and could be modularly customized depending on the virus. This strategy achieves efficient drug delivery and significantly kills 4T1 tumor cells based on good stability. In addition, the self-assembled product caused increased CRT exposure and a significant release of HMGB1, suggesting that the strategy enhances tumor immunogenicity by inducing ICD. The results showed that the tumor therapeutic effect of this nano vaccine was superior to that of conventional vaccines. Supramolecular peptides have better assembly properties and biosafety than proteins and small molecules. Wang et al. (78) designed and synthesized a TPA-FFG-LA nano vaccine by exploiting the self-assembly property of supramolecular peptides. The TPA-FFG-LA nanocomponent could play a disruptive role on lysosomal membranes, and the white light irradiation-induced ROS significantly exacerbated the Lysosome membrane penetration (LMP) that occurred. Nanovaccine-induced LMP can trigger ICD, reflected by increased CRT levels on the surface of 4T1 cells after vaccine treatment and a significant increase in secreted ATP levels. In a mouse model, TPA-FFG-LA achieved significant tumor suppression, and this therapeutic effect was based on a good biosafety profile.

The advantages of self-assembled nanomaterials based on nucleic acids, peptides, and proteins in inducing ICD include the following: (1) High biocompatibility: nucleic acids, peptides, and proteins are naturally occurring molecules in living organisms, and thus nanomaterials based on these molecules are usually characterized by good biocompatibility and low immunogenicity. (2) Precise targeted delivery: these nanomaterials can achieve high recognition and precise delivery of tumor-specific markers through molecular recognition mechanisms (e.g., antibody-antigen interactions, receptor-ligand binding, etc.). (3) Controlled drug release: By design, these self-assembled nanomaterials can responsively release loaded drugs under specific environmental conditions (e.g., pH, temperature, enzyme activity, etc.), thereby improving therapeutic efficiency and reducing toxicity to normal tissues. (4) Functionalization flexibility: self-assembled nanomaterials of nucleic acids, peptides, and proteins can be chemically modified or genetically engineered to introduce a variety of functional molecules, including immune activators, drugs, and imaging agents, to achieve multifunctional integration. (5) Mimicking pathogen characteristics: such nanomaterials can mimic the characteristics of pathogens to stimulate a strong immune response. For example, nucleic acid nanomaterials may be recognized as the genetic material of a virus, triggering an antiviral immune response in the host. (6) Tunability of self-assembly: the self-assembly behavior of these molecules can be modulated by changing conditions such as molecular design, concentration, temperature, etc., which enables precise control of the size, shape, and function of the nanomaterials.

### 3.2 Nanomaterials based on self-assembly of metal

In recent years, metal-organic framework nanomaterials formed based on the coordination self-assembly of metal ions have received increasing attention. Compared with typical inorganic nanoparticles and organic conjugated polymers, self-assembled metal materials have efficient drug-loading ability, easy modification, and good biocompatibility and can be used to build different nanoplatforms for functional enhancement by changing constituent units (79). Meanwhile, metal self-assembled materials are used for ICD induction, which provides a new research direction for metal self-assembly.

As TME-responsive therapeutic, diagnostic reagents, manganese dioxide nanoparticles have been widely used in cancer immunotherapy. Typically, MnO_2_-based nanomaterials can alleviate the hypoxic environment within the tumor by catalyzing the breakdown of hydrogen peroxide (H_2_O_2_) inside the tumor to produce oxygen to enhance cancer therapeutic efficacy. In addition to the above mechanism, applying MnO_2_ in combination with self-assembly is also becoming well-known. Yu et al. (80) designed MNFs (MnO_2_ nanoflowers) by self-assembly of KMnO_4_ with MES and enhanced stability using polyethylene glycol. The xenogeneic tumor transplantation model showed no effect of MNFs on cell survival. The MNFs were highly tumor-suppressive and prevented tumor growth and distant metastasis. The MNFs-treated cells showed high levels of CRT exposure and DAMPs levels, which provides a strong argument for the induction of ICD by nanoflowers. Notably, the team combined DMXAA with MNFs. This drug targets endothelial cells and disrupts the tumor vascular system to produce a cut-off of nutrient supply, thus eliminating the tumor. It showed a potent anti-tumor immune response in primary tumors with significant infiltration of CD8^+^ T cells. In short, this combined application enhances a new option for monotherapy, namely nanoparticle-coordinated cancer starvation immunotherapy, and is a typical reference for future monotherapies.

The importance of breast-conserving surgery for women cannot be overstated; however, the inability to determine negative margins during surgery leaves tumors intact, with the risk of recurrence, and a significant number of patients require multiple surgeries or even mastectomy, making it urgent to investigate accurate methods for determining breast cancer margins. There is a lack of relevant RT sensitizers. Gd has been shown to enhance the effect of RT in the presence of laser light. Based on this background, Zhang’s team (81) synthesized a Gd-based nanoprobe NPs-Bev. NPs-Bev is a spherical particle that remains stable in PBS, 10% FBS, and an acidic environment for a long time. In *in vitro* experiments, MDA-MB-231 cells were the most sensitive to the probe, with detectable probe fluorescence for a short period and significantly better uptake efficiency than other cells. Similar results were shown in tumor-bearing mice, where labeled NPs-Bev had a solid and persistent fluorescent signal at the tumor site. The team treated mice with NPs-Bev and then surgically excised the tumors under NIR-II guidance and found that there was essentially no residual signal in the remaining portion after excision. Compared with normal tissues, tumor tissues have stronger fluorescence signals and can be easily distinguished from normal tissues under NIR light. Besides, the efficiency of ROS generation under light far exceeded that of radiation alone. The combined high uptake efficiency brought about significant apoptotic results, which implied that the nanoparticles improved the killing effect of RT. The results showed that tumor growth was significantly inhibited. Overall, the probe not only provides precise guidance for the surgical resection of tumors but also enhances the RT effect, and the tumors can be removed entirely under the influence of the dual action, eliminating the possibility of recurrence. The construction of this multifunctional nanosystem based on metal Gd will be a significant advancement in molecular probes and promote the development of the field of molecular imaging, which is believed to be of great help in solving disease pains when applied in the clinic.

ROS can induce cells to undergo oxidative damage, which effectively induces ICD to increase cell immunogenicity. CDT-based Fenton reaction generates hydroxyl radicals by releasing Fe^2+^ efficiently combined with H_2_O_2_. Hydroxyl radicals can effectively induce ROS production and DC maturation, which implies that effective CDT can significantly trigger ICD. Ren et al. (82) used GOx (glucose oxidase) as a template to self-assemble with FeS to form FeS GOx nanoparticles while introducing PTX (paclitaxel) to construct FGP (FeS-GOx@PTX). GOx is a catalyst that can increase the intracellular H_2_O_2_ concentration and lower the pH value. In *in vitro* experiments, the concentration of H_2_O_2_ in FGP increased substantially with the addition of glucose. To obtain more substantial efficacy, the team combined PTT therapy. The PTT effect of FGP enhances GOx activity and promotes the production of hydroxyl radicals by elevating the temperature. Similarly, the same trend of enhanced Fenton response was shown in the 4T1 mouse tumor model, facilitating adequate ICD amplification to activate a robust anti-tumor response and inhibit metastasis. This work successfully constructed FGP by self-assembly, demonstrated enhanced efficacy of ICD, and provided a solution to tumor indiscriminate growth and metastasis.

Catalytic metal ions (Cu^2+^, Ce^4+^, Fe^3+^) can promote ^1^O_2_ production from lipid hydroperoxides, and the large amount of ^1^O_2_ production helps to alleviate the tumor hypoxic microenvironment. This new ^1^O_2_ generation mechanism can induce tumor cell death, and researchers are continuously developing and modifying it. Guan’s team (83) prepared LAHP (linoleic acid hydroperoxide) metal complex nanoparticles LAHP-M NP by ligand self-assembly of LAHP with transition metal ions (Cu^2+^/Fe^3+^) and then coassembled LAHP-M NP can dissociate into LAHP and metal ions under acidic environment. In *in vitro* experiments, LAHP-M NPs showed good tumor-selective properties and inhibitory effects, which were dependent on the concentration of nanoparticles. The peptide-modified NPs with the R7 sequence exhibited more potent inhibitory effects. Indeed, the massive production of ^1^O_2_ helped promote DC maturation, further facilitating the onset of ROS-mediated necrotic cell death. In addition, Xu et al. (84) designed and synthesized nanomaterials FIT NPs responsive to the TME using a supramolecular self-assembly approach. FIT NPs utilized fine coordination of Fe^3+^ to mix ICG with TAD for their co-delivery fully. The results showed that FIT had good stability, significantly increased ROS levels under laser irradiation, and induced desirable PDT effects. Besides, FIT NP could effectively induce ICD, which was derived based on the high level of HMGB1 and CRT expression. In the mouse colon cancer CT26 model, FIT NPs + Laser showed a therapeutic effect on tumors. In addition, FIT NPs + Laser contributed to DCs maturation and facilitated DCs cross-presentation of tumor antigens to CD8^+^ T cells to enhance anti-tumor immunity. In conclusion, FIT NPs trigger ICD to elicit a robust immune response and efficiently address the problem of sustained tumor growth, which deserves in-depth study for early application in clinical treatment.

Radiotherapy is a major therapeutic tool in the treatment of solid tumors. Its mechanism of action is mainly based on the damaging effect of high-energy radiation on cellular DNA, and it has also been found to induce the release of tumor antigens from the ICD and activate the immune system to attack tumors. However, insufficient deposition of X-rays in tumor tissues limits the ability of radiotherapy to generate sufficient ROS to induce ICD, so Yuan’s team (85) constructed AmGd-NPs based on the self-assembly of the high-atomic number metal gadolinium (Gd) and the small-molecule CD73 inhibitor AmPCP, which can effectively solve the problem of insufficient deposition of X-rays in the tumor. AmGd-NPs utilize the high atomic number metal gadolinium for radiosensitization, generating a large amount of ROS to induce ICD. In addition, AmGd-NPs can gradually release AmPCP, inhibit the enzymatic activity of CD73, and prevent the conversion of extracellular ATP to anti-inflammatory adenosine, thus creating a pro-inflammatory TME that promotes the maturation of DCs. The advantage of self-assembly is that it can effectively combine these two components, thus achieving synergistic effects of radiosensitization and immunomodulation and improving therapeutic efficacy.

Metal self-assembled nanomaterials-based nanomaterials offer several unique advantages in inducing tumor ICD: (1) Highly controllable and regulated: the size, shape, and surface functionalization of metal self-assembled nanomaterials can be precisely controlled by varying the synthesis conditions, which helps to optimize their biodistribution and tumor-targeting capabilities. (2) Enhanced photothermal/photodynamic therapeutic effects: some metal self-assembled nanomaterials, such as gold or silver nanoparticles, can effectively convert near-infrared light into thermal energy, triggering localized high temperatures and thus inducing ICDs. in addition, some metal nanomaterials can generate ROS, which enhances photodynamic therapeutic effects. (3) Catalytic activity: metallic nanomaterials may have catalytic activity, capable of catalyzing the production of ROS or other biologically active molecules *in vivo*, which can directly induce ICD or change the TME. (4) Imaging and optical imaging capabilities: self-assembled nanomaterials containing iron, cobalt, or other magnetic metals can be used as imaging contrast agents to provide tumor imaging and treatment monitoring. Also, certain metallic nanomaterials have unique optical properties that can be used for optical imaging. At the same time, equal attention should be paid to the potential drawbacks and challenges, including the lower biocompatibility of metallic materials as well as their degradability. Note also that while activating the immune system to fight tumors is beneficial, over-activation may lead to autoimmune diseases or other serious immune-related side effects.

### 3.3 Nanomaterials based on self-assembly of polymer

Polymers are polymeric compounds with relative molecular masses up to 10^3^-10^6^, promising tools for modification due to their stability, chemical diversity, and controllable molecular weight. Polymer self-assembly personalizes and improves drug targeting, efficiency, and other drawbacks by encapsulating several nanoparticles in the polymer bulk wall to obtain the corresponding morphology (86–88).

Based on the decisive role of DC cells in antigen presentation, many studies have focused on designing efficient DC vaccines to enhance the efficiency of immunotherapy. Nanotechnology provides a new platform for DC vaccine design, and based on the above background, Chen’s team (89) designed a multifunctional polymer CCPS/HPPH/DOX (CHD) formed by applying self-assembly technology to co-encapsulate DOX and HPPH within a multimeric PEG-P(MMA-co-AEMA(SH/NH_2_)-PDMA. CHD was essentially non-toxic to MC38 cells under laser irradiation, which undoubtedly showed that CHD is an efficient and safe drug delivery system. CHD-treated colon cancer cells exhibited adequate levels of DC maturation, CRT exposure, and HMGB1 release. Compared to PBS and DOX groups, tumor growth was significantly inhibited in CHD-treated mice. Under laser irradiation, CHD could exhibit more potent inhibition and prolonged survival in mice. In brief, the addition of multimeric nanomaterials stabilizes the entire system, improves delivery efficiency, triggers a more robust immune response, and has promising applications in clinical treatment compared to conventional agents.

Polymers are the new favorites for drug delivery due to their customizability, and polymeric micelles are one of the forms. Li et al. (90) developed an IND-based bifunctional immunostimulatory polymeric pre-drug carrier, which can self-assemble into nano micelles and deliver DOX. The polyethylene glycolization on the polymer surface reduces the adsorption of nanoparticles to serum proteins and increases the solubility of IND, which facilitates the improvement of drug delivery efficiency. The self-assembly polymers showed the most significant intra-tumor tissue damage and the lowest level of Ki-67 expression and a significant increase in CD4^+^ T and CD8^+^ T cells and a decrease in Treg cell numbers. Notably, this study reveals the superiority of polymeric nanomaterials, provides new insights into tumor treatment, and inspires researchers in general to study cancer therapies.

PDT is widely used for its low toxicity, aggressiveness, and ICD-inducing properties. However, the high lipidicity and poor pharmacokinetic response of most photosensitizers lead to their failure to achieve sound therapeutic effects. To address these issues, Wang et al. (91) designed and constructed redox-activatable liposomal RALs by self-assembling porphyrin phospholipid adducts and co-encapsulation of IDO inhibitors. This strategy exhibited high CRT and ICD-related DAMPs (e.g., ATP, HMGB1) in a 4T1 tumor-bearing mouse model. It induced significant ICD responses to promote the development of immune responses. *In vitro*, experiments to examine the phototoxicity of RAL showed that IND@RAL-based PDT promoted ICD in tumor cells and induced effective mitochondrial dysfunction and intrinsic cell apoptosis upon laser irradiation. After RAL injection into the 4T1 hormonal mouse model, the *in vivo* imaging system monitored a significant accumulation of RAL in the tumor, well above the adjacent muscle tissue. The RAL PPa pharmacokinetics showed a prolonged circulation time of RAL with an elimination half-life of 34.27 hours. The former showed significant tumor growth inhibition and tumor shrinkage rate as well as significant inhibition of tumor lung metastasis compared to control treatment with IND@RAL. In short, the above results provide a strong argument for the combined application of PDT-induced inhibition of the ICD-binding IDO pathway for tumor suppression and prevention of metastasis, laying a clinical translational foundation for developing new approaches and personalized tumor therapy.

Similarly, to address the problems of limited light penetration depth, the inefficiency of the immune response, and inefficient delivery of immunomodulatory drugs in PTT, Zhao et al. (92) designed a polymer self-assembled polymer-based nanomaterial, PMR NAs. PMR NAs consisted of NIR-II semiconductor Pdots with carboxyl group modification and R848 utilizing Mn^2+^ as ligand nodes. The advantage is that it enables autonomous drug release in response to the acidic conditions of the TME, which improves the therapeutic efficacy and reduces the toxic effects on normal tissues. This strategy utilized NIR-II light-excited photothermal therapy to achieve thermal ablation of deep tumor cells while releasing TAAs and DAMPs via ICD to stimulate immune response and induce tumor cell cuproptosis. Notably, this protocol utilized fluorescence imaging and photoacoustic imaging in the NIR-II region to achieve visualization of tumor tissue with deeper tissue penetration and higher imaging resolution.

In addition to the above, polymeric nano-self-assembly has many applications. Du’s team (93) designed a novel nanohybrid DNH (PEGylated pure drug-based nanohybrids) generated by self-assembling hydrophobic drugs OXA and 1-MT with hydrophilic polyethylene glycol (PEG). 1-MT is a clinically used IDO inhibitor that enhances immunotherapy by blocking immune resistance checkpoints. To enhance the tumor-homing ability of nanoparticles, the team prepared NK-DNH by coating NK cell membranes on DNH. Due to NK cell membrane artifacts, cell uptake increased compared to pre-treatment, which facilitated cancer cell targeting of nanomaterials. OXA can induce ICD, as reflected by CRT exposure and HMGB1 secretion within tumor tissue. CLSM images showed that NK DNH-mediated OXA exhibited the strongest CRT signal and significantly increased DC antigen (CD80^+^CD86^+^). The tumor growth inhibition and prolonged survival in mice brought about by NK-DNH are unquestionable.

**Figure 3:**
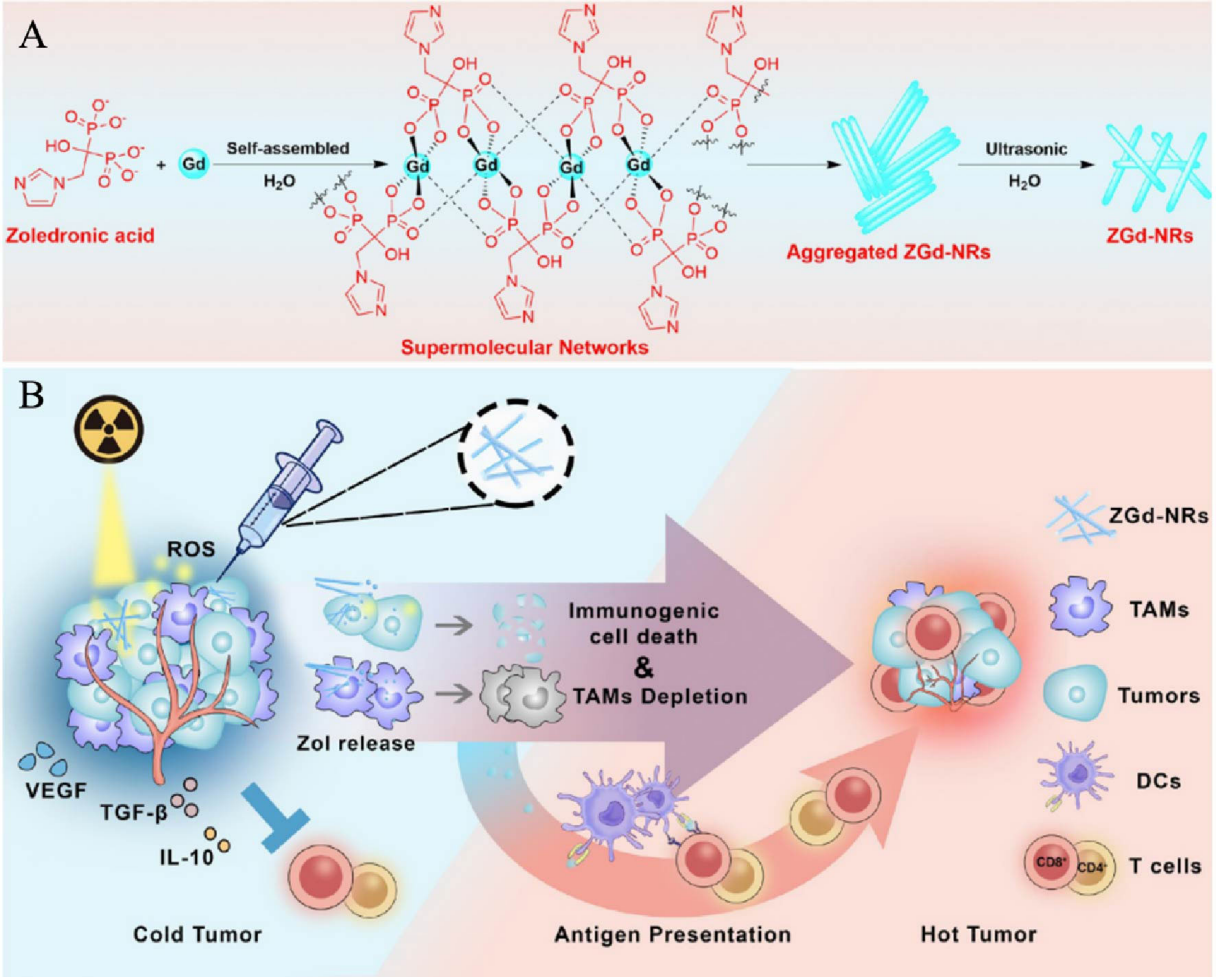
Synthesis and mechanism model of ZGd-NRs. **(A)**. Preparation diagram of ZGd-NRs. **(B)**. Mechanism of synergistic enhancement of tumor immunotherapy by ZGd-NRs inducing tumor ICD and TAM depletion (94). Copyright^©^ 2021, copyright Huang et al.

To reprogram the tumor immunosuppressive microenvironment, Yuan et al. (94) constructed self-assembled nanorods ZGd-NRs based on gadolinium (Gd^3+^) and zoledronic acid. ZGd-NRs can efficiently deposit X-rays and produce large amounts of ·OH, which facilitates the induction of immunogenic death of tumor cells (**Figure 3**). In the CT26 tumor model, ZGd-NRs showed some blocking effect. When combined with RT, tumor cells were significantly reduced, triggering more CRT exposure and more ATP and HMGB1 release in CT26 cells. Synergistic treatment of ZGd-NRs with RT resulted in reduced levels of immunosuppressive factors in the TME, represented by TGF-β1, IL-10, and VEGF-A. These results suggest that nanoparticles deplete tumor-associated macrophages to revert into an tumor immunosuppressive microenvironment(TIME), enhancing anti-tumor immunity. In *in vivo* experiments, the infiltration rate of CD4^+^ T and CD8^+^ T cells was significantly higher in the ZGd-NRs + RT group, suggesting that the combined application promoted the immune response. Meanwhile, the 150-day survival rate of the ZGd-NRs + RT group was 60%, significantly higher than all other groups. These experimental results reveal the significant effect of ZGd-NRs on tumor growth inhibition and prolonged survival and also provide a classic example for developing new pathways in tumor therapy, to design more anti-cancer therapies based on polymeric nanomaterials in the future.

To address the problems of limited light penetration depth, inefficacy of immune response, and inefficient delivery of immunomodulatory drugs in PTT (95–97), Shen et al. (98) designed a polymer self-assembled nanomaterial based on PMR NAs.PMR NAs consist of NIR-II semiconducting Pdots (polymer dots) with carboxylate group modification and immunomodulators (R848) to induce ICD in tumor cells using manganese ions (Mn^2+^) as ligand nodes to induce ICD in tumor cells by releasing DAMPs. The advantage is that autonomous drug release can be achieved in response to the acidic conditions of the TME, thereby improving therapeutic efficacy and reducing toxic effects on normal tissues.

Polymer self-assembly-based nanomaterials have a wider range of applications in inducing ICD in tumor cells, including (1) Tailorability: the composition and structure of the polymers can be adjusted by chemical synthesis methods to optimize their biodistribution, drug loading, and release kinetics. (2) Synergistic therapy: polymeric nanomaterials can be used in combination with other therapeutic approaches (e.g., photothermal therapy, radiotherapy, etc.) to enhance therapeutic efficacy and immune response. However, the biocompatibility and toxicity of these nanomaterials should not be overlooked. Although many polymers are considered biocompatible, the long-term stability and potential toxicity of the nanomaterials need to be further studied and evaluated. Their development should be based on the collaboration of several fields, including oncology, materials science, immunology, and pharmacology, to jointly develop and optimize new therapeutic strategies.

The application of self-assembled nanomaterials in inducing ICD for therapeutic purposes is a rapidly growing field, showcasing the versatility and potential of these nanomaterials. However, the scalability and manufacturing challenges associated with these technologies vary depending on the type of self-assembled nanomaterials.

Self-assembled nanomaterials derived from nucleic acids, peptides, and proteins offer high specificity and biocompatibility. They can be finely tuned to target specific cellular components and pathways, making them highly effective for therapeutic ICD induction. However, the scalability of producing these materials faces significant hurdles. The synthesis and purification processes can be labor-intensive and costly, requiring stringent conditions to maintain the integrity and functionality of the biological molecules. Additionally, large-scale production needs to address variability in molecular self-assembly, ensuring consistent quality and performance.

Metal-based self-assembled nanomaterials, exhibit strong plasmonic properties and can be functionalized to target cancer cells specifically. They are potent inducers of ICD due to their ability to generate ROS and heat upon irradiation. The manufacturing of metal-based nanomaterials is relatively scalable, as chemical synthesis methods can be adapted to larger volumes. However, ensuring uniform particle size and surface modification at scale can pose challenges. Additionally, the biocompatibility and potential toxicity of metal nanoparticles need thorough assessment during the manufacturing process.

Polymer-based self-assembled nanomaterials are highly versatile, allowing for the incorporation of various therapeutic agents and functionalities. They offer controlled release profiles, enhanced stability, and the ability to design stimuli-responsive systems that specifically release drugs in the tumor microenvironment. While the scalability of polymer synthesis and self-assembly processes is relatively well-established in the industry, there are still challenges in achieving uniformity and reproducibility of the nanomaterials at scale. The complexity of polymer chemistry and the need for precise control over molecular weights and polydispersity can impact the consistency of production.

In summary, self-assembled nanomaterials encompass a diverse range of systems, each with unique benefits and limitations in inducing ICD for cancer therapy. While nucleic acid, peptide, and protein-based nanomaterials offer high specificity and biocompatibility, they face significant scalability challenges. Metal-based nanomaterials demonstrate potent ICD induction capabilities but require careful management of particle uniformity and biocompatibility in large-scale production. Polymer-based nanomaterials provide versatility and controlled release options, yet achieving consistent quality at scale remains a challenge. Addressing these manufacturing hurdles is crucial for the successful clinical translation and widespread use of self-assembled nanomaterials in cancer therapy.

## 4 Metallic materials

Metallic elements are essential components of the living body and play a role in almost all life processes. Many new products have been born in nanomaterials that combine metallic elements with traditional therapeutic methods (99–102), and these products use the action of metallic elements in living organisms to improve the defects of original therapeutic methods, such as Zhang et al. (103) who combined quercetin with iron ions to improve PTT therapeutic effects by modulating the immunosuppressive microenvironment. There are many more such examples, and based on the great promise of metal nanomaterials, this section will review the applications based on metal particles, metal ions, and metal MOF in tumor ICD.

### 4.1 Metal compounds-based materials

Metal elements are widely used in daily life, not only as atoms and ions but also as a variety of compounds, such as oxides, sulfides, complexes, and other types. Different metal particles have different functions. Usually, metal compounds have high melting points, hardness, and brittleness and cannot be applied directly (104, 105). In living organisms, metal compounds are often involved in metallic processes. Therefore, altering metal compounds may have therapeutic effects on diseases. The emergence of nanotechnology has dramatically enriched the clinical applications of metal compounds, and some scientists have proposed various tumor treatment options in combination with metal compounds.

PD-1/PD-L1 checkpoint blockade immunotherapy has received much attention for inducing regulatory T cell-induced immune responses. However, low immunogenicity greatly hinders the aggressiveness of PD-1/PD-L1 therapy, and an effective strategy is to enhance immunogenicity by inducing ICDs in cells. Based on this study, Yan’s team (106) synthesized the phenolic ICD inducer MDP NP by self-assembling DOX, phenolic manganese dioxide nanoreactors, iron, and PEG polyphenols via metal-phenol ligands. It was shown that increasing intracellular oxygen concentration or ROS, content contributed to enhancing DOX-based ROS-dependent cell death. Fortunately, MnO_2_ nanoparticles could assist in intracellular H_2_O_2_ catabolism. Under acidic conditions, MDP NP exhibits excellent H_2_O_2_ nanocatalysis ability, allowing for rapid O_2_ production. This facilitates the alleviation of intracellular hypoxia and enhances ICD effects. Within B16-F10 tumor-bearing mice, MDP NP treatment leads to a robust ICD response, as evidenced by significant CRT exposure and HMGB1 production, promoting antigen production. MDP NPs showed good tumor growth inhibition very early after the treatment of mice, such that the therapeutic effect was based on the advantages of prolonged drug circulation time and high tumor accumulation. Excitingly, the combination treatment of the self-assembly product with PD-1 checkpoint blockade immunotherapy significantly induced apoptosis and necrosis in most tumors. Overall, the success of this study is attributed to the decomposition of metallic manganese dioxide to produce O_2_ and the combination of αPD-1 therapy, and these research foundations hold promise for the use of metallic nanomaterials for clinical applications and treatment of human tumors.

Studies have shown that NIR-II is gradually replacing NIR as a light source for PTT due to its excellent penetration and ability intensity (107–109). The STING (stimulator of interferon genes) is a crucial signal transduction molecule in the participant immune response, which promotes DC maturation, antigen presentation, and activation of T cells (110). Based on the above background, Lu et al. (111) constructed a nanosystem dNAc/NIR-II that activates the intracellular STING pathway. dNAc is composed of heat-responsive liposomes encapsulated with iron sulfide nanoparticles (FeS_2_) and cGAMP, followed by ECM degrading enzymes. FeS_2_ nanoparticles act as photothermal converters and catalysts for the Fenton reaction, which can jointly participate in CDT and PTT treatment. The nano-agonist dNAc generates highly toxic -OH by reacting with H_2_O_2_, significantly reducing cancer cell activity, which can be further reduced after NIR-II laser treatment. Meanwhile, the nanosystem-treated 4T1 cells exhibited adequate CRT levels while releasing large amounts of HMGB1 extracellularly, suggesting that dNAc/NIR-II enhances CDT-induced ICD. In addition to the increase in DAMPs observed in the *in vivo* experiments, an increase in the number of DCs maturing under the influence of the nanosystem could be detected, up to 1.96 times that of the saline control. The researchers went on to treat tumor-bearing BALB/c mice with the nanosystem, which showed more minor tumor levels and played an integral role in inhibiting tumor metastasis. In short, this study demonstrates the effectiveness of an ECM-degrading nano-agonist that utilizes a mild NIR-II photothermal binding to the STING pathway for enhanced cancer CDT immunotherapy efficacy.

As research progresses, nanomaterials are increasingly being used directly or indirectly in the anti-cancer field with promising chemical effects. However, TME can build a series of “walls” that prevent the drug or mechanism from working, such as inhibitory environments and barriers to easy access. Based on the finding that inhibition of IDO1 expression has a positive impact on improving immunosuppression (112–114), Feng’s team (115) proposed a nanomaterial DNCaNPs combining DOX, alkylated NLG_919_ (aNLG_919_), and CaCO_3_. The researchers found that the release efficiency of DOX was acid-dependent, decomposing 49.3% at pH 5.5 in only 24 h. However, the release rate of aNLG_919_ was slower, probably due to the easy acid-binding nature of CaCO_3_. In both *in vivo* and *in vitro* experiments, the DNCaNPs effectively released DOX to produce anticancer effects compared to the control and other experimental groups and significantly inhibited IDO1. Notably, the efficient release of ATP, HMGB1, a signature factor for ICD triggering, was detected during both treatments. DNCaNPs were seen to inhibit CT26 tumors in animal experiments and prolong the survival of mice. Notably, in addition to the subcutaneous CT26 colorectal tumor model, DNCaNPs also exhibited therapeutic effects on 4T1 tumors *in situ*, which is surprising and implies that the applicability of the nanoformulation is not unique. In summary, this study uses the properties of CaCO_3_ to modify the original experimental approach to act on three aspects: loading capacity, delivery efficiency, and improved immunosuppression, and ultimately to promote DC activation to elicit anti-tumor immune responses. Overall, this strategy provides an excellent example of innovative and diverse tumor research, and inspired by this, we can also address the challenges in future work by fine-tuning the basic approach.

Magnetic iron oxide nanoparticles have excellent physical properties and can exhibit superparamagnetism at specific temperatures. This property allows iron oxide nanoparticles to be used in various applications such as diagnostics, MRI contrast agents, magnetic thermotherapy, and drug delivery (116, 117). Based on the above research basis, Li et al. (118) built a nanoplatform SPIOs@PLGA@Au nanoparticles (DSG NPs) characteristically targeting Her-2 breast cancer by a series of methods using superparamagnetic iron oxide nanoparticles (SPIOs). The characterization analysis of the nanoplatform revealed that DSG NPs could pack a specific content of DOX and SPIO nanoparticles, accounting for 3.1 ± 0.66% and 2.88 ± 0.11% of DSG, respectively. And with increased iron oxide nanoparticle content, DSG had higher contrast enhancement in T2-weighted MRI. *In vitro* experiments showed that DSG exhibited the expected cancer cell targeting, and the results indicated that the cancer area showed a sizeable dense fluorescence after DSG irradiation, which implied that DSG could enter the cancer cells in large quantities and then act. BT474 was an HER-2-infected tumor cell, and HSP70 was on its surface.

The data showed that the amount of HSP70 and CRT on cancer cells increased with the duration of drug-cell contact, suggesting a possible metal particle-catalyzed cause and an outcome-induced ICD (119, 120). After intravenous DSG injection, the researchers performed The tumor volume of laser-illuminated DSG NPs-treated mice was significantly reduced (mean shrinkage to 8.19 ± 5.05%) compared with all other groups, and the treatment effects were based on good biosafety. This study fully illustrates the positive nature of photothermal-induced therapeutic tools with clinical treatment prospects. Similarly, Cui’s team (121) prepared a nanoprobe system Fe_3_O_4_@MnO_2_-celastrol (CSL)/Ce6 based on manganese and iron compounds. The probe can induce H_2_O_2_ decomposition, facilitating oxygen generation and alleviating hypoxic limitation. In addition, the nanoprobe was readily taken up by cancer cells and detected red fluorescence in the cytoplasm, which could be attributed to the EPR effect of the nanomaterials. In both *in vivo* and *in vitro* assays, the nanoprobes rapidly reached the tumor and efficiently promoted cancer cell apoptosis through PDT and mediated ICD. Such superior imaging and therapeutic effects were established by targeting only tumor cells, and normal tissues and organs were unaffected. In summary, this probe solves the dilemma of poor targeting of traditional tumor drugs and the inability to accurately diagnose and observe the therapeutic effect. It has excellent clinical translational value in precise diagnosis and treatment.

### 4.2 Metal ions-based materials

Metal ions occupy a vital position in the research in the life sciences. For example, Mg^2+^ is involved in protein synthesis and almost all energy metabolism, oxygen transport by iron ions, and regulation of signaling by Ca^2+^ (122–124). With the rise of ICD treatment, the role of metal ions in combination with traditional methods to enhance tumor immunotherapy has also been identified and intensively studied by researchers, based on which various new strategies have been proposed to improve the efficiency of cancer treatment effectively.

CFZ (Carfilzomib) is a newly discovered chemotherapeutic agent that can trigger ICD and is gaining attention in the pharmaceutical field. However, high doses of chemotherapeutic agents produce killing effects while damaging immune cells, making the induced ICD insufficient to trigger a robust immune response again (125, 126). Therefore, reducing the dose and enhancing the efficacy has become a pain point for many researchers. Taha et al. (127) noting that nanomaterials have recently been heavily used, constructed a nanosystem CFZ-pTA-alb that can induce ICD through a two-step reaction composition. First, TA (tannic acid) self-assembled with Fe^3+^ to form a shell and loaded CFZ to obtain a stable and homogeneous spherical CFZ-pTA; then, the end product CFZ-pTA-alb was obtained by modifying the spherical structure with albumin, which exerted a blocking effect and slowed down the drug release. Compared to free CFZ, low doses of CFZ-pTA-alb can release the drug slowly and continuously, and eventually, the same drug concentration is detected in cancer cells. In addition, the nanosystem has an antigen adsorption and delivery function, which can capture TAAs with DAMPs delivered to DCs, which may be the role of pTA. In animal experiments, tumor growth was significantly inhibited after CFZ-pTA-alb treatment, the CFZ content in the tumor was detected to be very significant, and DCs and lymphocytes were retained in large numbers. Notably, the team also constructed a distant tumor model, and the gel unsurprisingly activated systemic T-cell immunity to harm distal tumors, thereby limiting tumor growth. Overall, CFZ-pTA-alb addressed the limitation of ICD by high dose use of CFZ and obtained higher intratumoral drug accumulation and enhanced ICD response by slow and sustained drug release. It also provides an idea for future work. Whether this mechanism can be applied to other chemotherapeutic agents, and it is foreseeable that if this protocol becomes widespread, it will be less costly and less physically and mentally stressful for patients.

Studies have shown that specific polyphenols can positively affect cancer and many diseases through their antioxidant effects. Also, natural polyphenols can easily bind to metal ions to form metal phenol network carriers with cargo-carrying effects. Based on the above research, Shen’s team (128) used DACHPt to complex with natural polyphenols to form metal-organic ligand nanoparticles. Meanwhile, Fe(III) was cross-linked with polyphenol-Pt(II) to obtain structurally stable and acidic environment-sensitive nano-TA-Pt/Fe (PTI NPs) in an attempt to achieve efficient OXA delivery via PTI. Excitingly, the PTI NPs exhibited the expected tumor cytotoxicity and reached the strongest at pH=5.0. In addition to this, both TA and COX increased cellular secretion of ATP levels and CRT exposure. Also, they contributed to the extracellular release of HMGB1, mechanisms that were established in an acidic environment (pH=5.0). Flow analysis yielded that PTI NPs under acidic conditions actively promote DC maturation (up to 36%) and reduce Treg levels (68% of free OXA), which contribute to tumor antigen delivery and anti-tumor immunity, and are an indication of ICD occurrence in tumor cells. After intravenous injection of PTI NPs in mice, the data showed that PTI rapidly assembled tumor areas and retained them for a considerable period. This study identified the decisive role of metal-polyphenol structure in cancer therapy as a promising therapeutic strategy.

Lactic acid, a product of glycolysis in the body, has also been found to aid tumor cell immune escape and promote development *in vivo*. Studies have shown that lactic acid can negatively affect many immune cells (e.g., macrophages, DCs, effector T cells) (129–131). Traditional nano approaches to breaking down lactic acid by introducing new drugs can lead to complications in nano design. To address this issue, Li et al. (132) chose cationic polyethyleneimine as a carrier and inserted LOX (lactate oxidase) with copper ions to obtain the nano vaccine PLNPCu. Observational experiments in morphology showed that the neutral mitigation nano vaccine presented a PEG-coated dispersed homogeneous spherical structure. Under weakly acidic conditions (pH=6.5), PEG can detach to form a shell-less structure to facilitate the reaction. The researchers measured the ability of LOX to consume lactic acid. The PLNPCu group had a much higher lactic acid adsorption rate of 19.54% ± 0.559 than all other groups, indicating that LOX plays a complete lactic acid recruitment role in the nanosystem. Lactic acid catabolism produced large amounts of H_2_O_2_, which mixed with Cu^2+^ to produce vital cytotoxic ·OH, leading to apoptosis of tumor cells. The ·OH produced by the Fenton reaction in which Cu^2+^ participated could further increase the level of ICD markers to promote ICD-induced death of tumor cells. PLNPCu promoted CD80 and TNF-α expression and inhibited CD206 and Arg-1 expression to induce macrophages to maintain the M1 polarization state to enhance the anti-tumor immune response. In short, this study effectively combined CDT with metabolic therapy to perform powerful functions to address the adverse effects of lactate on the immune response, and this approach to eliminate the effects deserves to be applied by us in other experiments.

Zhang et al. (103) used an ethanol injection method to mix quercetin and iron ions to obtain phenolic metal nanoparticles QFN with photothermal effect, a facilely synthesized nanostructure with 42.1% photothermal conversion efficiency. It was shown that quercetin enhanced the anti-tumor activity of CTLs by affecting PD-L1 through inhibition of the JAK2/STAT3 pathway. Meanwhile, QFN-PTT triggers tumor ICD, which further promotes the maturation of BMDCs and thus releases more functional cytokines. In addition, QFN captures the antigen by adsorbing the melanoma antigen tyrosinase-related protein 2 and other targets the resulting antigen to lymph nodes. *In vivo* experiments showed that the QFN-PTT-treated B16F10 melanoma mouse model resulted in complete tumor killing and no recurrence. Other tumor re-attack experiments showed that TNFα, IFNγ, and IL-6 were increased several-fold in the cured mice compared to the control group, resulting in short-term tumor-free and 1/3 tumor-free after 100 days, which far exceeded the researchers’ expectations. More excitingly, the QFN in this study positively affected the anti-tumor immune response in various models, including primary melanoma/mammary tumor models, tumor recurrence models, contralateral tumor models, and metastasis models. To reverse the TIME to improve the efficiency of ICB therapy, Ge’s team (133) developed a ROS-responsive nanoformulation of P/G@EFTKNPs, which first utilizes OEGCG as a binder to compound αPDL1/GOx, which in turn binds Fe3^+^ and is covered with a block copolymer POEGMA-b-PTKDOPA composition. After intravenous injection, the nano-formulation can be aggregated around the tumor using the EPR effect. Upon entry into the body, Gox in the complex retains some of its activity to generate H_2_O_2_ and reduce Fe^3+^ to Fe^2+^, promoting the generation of large amounts of ·OH from H_2_O_2_, leading to the breakdown of the complex. P/G@EF-TKNPs inhibited primary tumor growth and effectively suppressed distant tumors within 3 weeks. Notably, the levels of CD8^+^/CD4^+^, TNF-α, and CTLs were increased to different degrees after P/G@EF-TKNPs treatment, suggesting that the tumor immune microenvironment may change from immunosuppressive “cold” to immunogenic “hot”. This suggests that the tumor immune microenvironment may change from immunosuppressive “cold” to immunogenic “hot.” All experimental data indicate that ROS-responsive nanomaterials provide a nanoplatform for combined CDT and ICD treatment, showing superiority over conventional approaches in tumor treatment.

### 4.3 MOFs-based materials

MOFs (metal-organic frameworks) are porous materials synthesized from metal ions or metal clusters with organic ligands using direct encapsulation, self-assembly, and post-modification methods. Thanks to the advanced compositional elements and synthesis methods. MOFs possess a solid ability to load metal ions and metal elements, high porosity, structural diversity, and excellent biodegradability (134, 135). These advantages make MOFs an ideal platform for drug delivery and cancer treatment.

As research progresses, more and more oncology treatment options are being discovered and discussed. Due to drug resistance, standard treatment options such as DOX gradually lose their expectation. At this time, a new metal-organic backbone material that can treat the disease simultaneously in many aspects has set off a new wave of cascade amplification for cancer treatment. In this context, Shen et al. (136) constructed metal-organic skeletal materials (SS) MOFs based on the interaction of Fe^3+^ with disulfide ligands. This also makes the nuclear spherical structure of mFe(SS)/DG more uniform and stable. In *in vitro* experiments, GOx-catalyzed production of large amounts of H_2_O_2_ increased intracellular ROS levels, as did disulfide-induced GSH depletion. mFe(SS)/DG caused ferroptosis in tumor cells by downregulating GPx4 (Glutathione peroxidase 4) expression, enhancing LPO accumulation, and generating large amounts of ROS to inhibit glycolysis. This led to a reduction in lactate production and facilitated the re-programming of TIME. MTT assays showed that mFe(SS)/DG exhibited superior cancer cytotoxicity than other groups due to the synergistic effect of ferroptosis and DOX. Also, this synergistic effect significantly enhanced free DOX-induced ICD capacity by further promoting DC maturation and DAMPs production. After each group was treated with the *in situ* 4T1 model, mFe(SS)/DG treatment demonstrated the most decisive effect expected due to homologous targeting and the combination of ROS-ferroptosis-glycolysis with chemotherapy, with treatment results showing the most severe cancer cell destruction and lowest systemic toxicity in this group. Based on these good performances, the cascade amplification treatment approach was feasible and will provide future innovations in oncology treatment.

Porphyrin-based MOFs are widely used for their high crystallinity, large porosity, and inherent photoactivated ROS generation among various PDT nanomaterials. Zeng’s team (137) utilized porphyrin zr-based MOF (porphyrin Zr-based metal-organic framework) combined with lanthanide NaYF4:Gd, Tb@NaYF4 scintillation nanoparticles (SNPs) to generate a novel soft x-ray nanoprobe SNPs@ZrMOF. Soft X-ray irradiation resulted in the most significant decrease in absorbance (61%) of the DPBF solution containing SNPs@ZrMOF, indicating that SNPs@ZrMOF has a high ROS generation efficiency. The nanoprobe combined with X-ray treatment of 4T1 tumor-bearing mice showed cancer cell killing ability consistent with expectations, and such therapeutic effect was based on good biocompatibility and homologous targeting. Besides, the process of PDT triggering ICD was also enhanced by SNPs@ZrMOF, as shown by the higher CRT expression and HMGB1 content than all other groups, which played a prominent role in promoting anti-immunotherapy induction. Also, under the influence of nanoprobe and light, IL-6 and TNF-α expression was elevated, indicating that it modulated immunosuppression TME. Overall, SNPs@ZrMOF exhibited significant pro-tumor apoptosis and improved TME, and we believe such drugs have a high potential for clinical translation.

In recent years, scientists have created combination therapy regimens that maximize the potential to improve the efficiency of cancer treatment. The discovery of nano-MOF can become a carrier for tumor therapy and has triggered many research teams to innovate multiple synergistic therapeutic regimens based on MOF. Based on MOF, Wen et al. (138) put ICG and DOX to construct a nano-agent MOF@ICG@DOX (DIMP). ICG (indocyanine green) is a widely used contrast agent for precise diagnosis and treatment of cancer. Still, the liver efficiently clears it due to its low circulation rate in the body. dIMP effectively solves this problem with the properties of MOF. DIMP has a 90.7% release rate of DOX at pH=5. Acid-sensitive DIMP can effectively break down the chemotherapeutic drugs in TME and improve efficacy. In *in vitro* experiments, DIMP under light has an efficient ability to generate ROS, and the experimental results of the DIMP + Laser group showed surprising mean fluorescence intensity values (≈13), supporting this conclusion. DIMP + Laser treatment after laser irradiation produced significantly high levels of CRT and HMGB1 expression in both cells and blocks, i.e., promoting ICD development in cancer cells. PA imaging results showed that DIMP was enriched in tumor sites after injection into mouse tumor models, a surprising finding. In summary, this DIMP-like treatment protocol combining chemotherapy/PDT/PTT under multiple imaging guidance is superior and provides a powerful approach to cancer treatment in the clinic.

In recent years, ICB and sonodynamic therapies have been hot spots for treating tumors. Still, they inevitably have the disadvantages of inadequate antigen presentation, low efficiency, and high metabolism (139–141). Careful researchers speculated whether an nMOF could be applied to complement their strengths. Huang’s team (39) piggybacked on the TPP-coupled porphyrin-based nMOFs product Zr-TCPP(TPP) NPs for enhancing SDT treatment efficiency to achieve a robust anti-tumor response. The researchers also modified nMOFs with 4T1 cancer cell membranes, combining R837 as an immune adjuvant to generate Zr-TCPP(TPP)/R837@M. This nMOF has the advantages of homologous targeting ability and high toxicity in cancer cells. Meanwhile, this nanomaterial could effectively promote the exposure of CRT in the 4T1 tumor cell membrane. At the same time, the release of HMGB1 from the nucleus was detected most significantly after 1 min of US irradiation, confirming that Zr-TCPP(TPP)/R837@M could effectively induce ICD in tumor cells, and promote DC maturation and immune-related cytokines (IL-6, IL-12p40, and TNF-α, etc.)*in vivo*. However, Treg cell content in tumors increased significantly after these treatments, inhibiting ICD. To address this issue, Huang et al. blocked CTLA-4 activity using immune checkpoint blockade, and the combined treatment showed enhanced anti-tumor efficiency and tumor metastasis inhibition. To promote tumor antigen presentation and infiltration of DCs, Zhou’s team (142) synthesized the photothermal nano vaccine PDM based on poly(5 -hydroxytryptamine) core and TA/Mn^2+^ ligand inserted into nMOF. This nano vaccine could generate a powerful photothermal effect and induce ICD for antitumor effect after microneedle insertion. In *in vivo* experiments, the PDM-treated melanoma mouse model promoted DC maturation by increasing CRT exposure after phototherapy, leading to more antigens being released and delivered. Meanwhile, PDM further recruited T cells to participate in anti-tumor immunity by down-regulating β-catenin levels and increasing CCL4 content. The results showed a significant reduction in tumor volume and weight in the PDM group, while the efficacy aligned with expectations was also based on essentially no skin damage. These findings have greatly encouraged our confidence in treating cancer and will promote the clinical application of nMOF as a new strategy for anti-tumor immunotherapy.

When using metal-based nanomaterials to induce ICD in tumor, potential toxicity and safety issues are critical considerations. Despite the immense potential of metal nanoparticles in medical applications, their associated biosafety issues pose significant challenges for clinical use. Metal nanoparticles can significantly increase the levels of ROS within cells under certain conditions, inducing cellular stress responses, oxidative damage, and even apoptosis or necrosis. While moderate increases in ROS are beneficial for inducing ICD, excessively high levels can lead to unnecessary tissue damage and cellular toxicity. Due to their small size and difficulty in metabolism, metal nanoparticles tend to accumulate in specific tissues and organs within the body. This accumulation can potentially trigger chronic inflammatory responses or other negative health effects, resulting in potential long-term toxicity. Different types of cells exhibit significant variability in their responses to metal nanoparticles. Some cell types may be highly sensitive to these particles, which means that in physiological environments where multiple cell types coexist in close proximity, metal nanoparticles may induce nonspecific toxic reactions. Regulatory considerations for clinical use include stringent safety evaluations before metal nanomaterials can be applied clinically. These evaluations comprise acute and chronic toxicity tests, *in vitro* and *in vivo* immunogenicity tests, and detailed biodistribution studies. Implementation of long-term monitoring and follow-up studies is crucial to assess the long-term safety and efficacy of metal nanoparticles in clinical applications. This includes tracking potential delayed toxic reactions and possible chronic health effects. Ensuring high consistency and safety of metal nanoparticles during commercialization requires standardized manufacturing processes and strict quality control. The production processes should be rigorously regulated, from the selection of raw materials to the final product packaging, with all stages adhering to Good Manufacturing Practice standards.

In summary, metal-based nanomaterials show significant promise in inducing therapeutic ICD, but their potential toxicity and safety issues cannot be overlooked. By optimizing design, surface modification, dosage control, and rigorous regulatory evaluations, these risks can be gradually mitigated, making the application of metal nanoparticles in clinical treatments safer and more effective.

## 5 Mesopore materials

Immunotherapy that induces ICD in tumors can effectively inhibit both *in situ* and distant tumor growth and is a cancer treatment that is being intensively investigated. Traditional ICD inducers can work but with limited effect, so inducers with more substantial effects and more comprehensive cancer selection are of great research value. Based on this premise, some scholars have found that mesoporous materials as drug carriers can improve the regimen’s efficacy. Mesoporous materials are 2-50mm pore size, featuring high specific surface area and modifiability with large pore size (143–145). This section classifies mesoporous materials in tumor immunotherapy using the classification criteria of CDT, PDT, PTT.

### 5.1 CDT nanomaterials

The most important thing for tumor immunotherapy is to discover the “cradle” to block the rapid growth of cancer and build products based on it that are easy to synthesize, have few side effects, and are powerful. Lin et al. (146) found that MnOx NSs (MnOx Nanospikes), which are easy to prepare and have mesoporous structures, can be loaded with antigens in large quantities and play a corresponding role in ICD therapy. *In vitro* experiments showed that MnOx decomposed Mn^2+^ to generate large amounts of ·OH, which was toxic to tumor cells and exerted a standard CDT effect. In addition, the researchers found a decrease in GPx4 protein content and a deepening of LPO (lipid peroxidation), suggesting that the nanodrug can also lead to cellular ferroptosis. In the presence of both mechanisms, tumors triggered ICD through cell surface CRT exposure, HMGB1 release, and massive ATP production. Mesoporous nano treatment of the 4T1 mouse tumor model showed a peak in CD8^+^ T content, thoroughly stimulating tumor immunosuppression. Surprisingly, the mice also developed immune memory and showed faster and stronger clearance after the second vaccination. In addition to the proud therapeutic effect of the procedure, MnOx was also found to show vigorous PA and MR signals after injection, which can be applied to dual-mode imaging of MR/PA. Overall, the team found for the first time that MnOx can be used for ICD induction with a significant positive impact. The above study demonstrates effective damage to tumors, and this work broadens the application of MnOx nanomaterials and deepens the understanding of nanomaterials, which will undoubtedly lead to the wave of nanomaterials for disease treatment.

Wang’s team (147) constructed a biodegradable gold nanoparticle Au@HMnMSNs, an elliptical structure constructed by doping manganese into gold core-mesoporous silica. The team then used PEG to modify and load ASA (aspirin) and DOX to obtain DOX/ASA@PEG-Au@HMnMSNs. ASA was shown to downregulate Cox-2, which is the “bridge” that determines whether DC cells can pursue tumors. The advanced mesoporous structure shows remarkable biodegradation ability, especially in acidic and high GSH environments, which rapidly releases DOX and ASA and exerts a GOx-like enzyme reaction to enhance H_2_O_2_ concentration. The cytotoxic ·OH is produced in large quantities with the release of GSH and H_2_O_2_ and the release of Mn^2+^. In *in vitro* experiments, PEG-Au@HMnMSNs can rapidly accumulate at tumor sites within a short period and remain there for a considerable period. The Gox-like response triggers ICD by upregulating CRT and HMGB1 expression, stimulating DC cell maturation and antigen presentation. This further leads to increased CD8^+^ T and CD4^+^ T content in tumors and promotes tumor killing by CTLs. After drug treatment of 4T1 hormonal tumors, the results showed extensive tumor necrosis and a significantly higher apoptosis rate than the control group. Encouragingly, normal tissues of mice were unaffected under such a powerful killing effect, indicating that the drug was safe. In conclusion, this work induces an immune response triggered by ICD by constructing a mesoporous nanocarrier to deliver ASA and DOX to the tumor target efficiently and safely. And it draws on the Gox enzyme response to amplify the CDT effect and effectively inhibit tumor growth.

Shao et al. (148) used this property to construct an ICD amplification system MON@KP1339 by combining diselenide-modified MONs (mesoporous organic silica nanoparticles) with KP1339. KP1339 is a chemotherapeutic agent shown to induce an ICD-primed immune response. Due to the combined effect of selenium and ruthenium, the system could efficiently reach the target and rapidly release KP1339. Meanwhile, MON@KP1339 exhibited up-regulated levels of CRT, HMGB1, and ATP compared to KP1339 alone, and interestingly, this ICD-enhancing effect showed a MON carrier concentration-dependent trend. In addition to the enhanced expression of DAMPs, this system also consumed a large amount of intracellular GSH to generate ROS, which is exactly in line with the mechanism of high ROS killing by selenium-leading tumors. MON@KP1339 was also confirmed by the increase of cytokines such as TNF-α. CM@MON@KP1339 with selective endocytosis was obtained by modifying the system with the cell membrane of 4T1 cells to enhance the tumor-targeting ability further. The team constructed 4T1 *in situ* mammary ruffled mice for *in vivo* experiments and showed that CM@MON@KP1339 exhibited significant tumor effects on *in situ*, distant and metastatic tumors. Similarly, Yu’s team (149) constructed a nanodrug based on PMO (periodic mesoporous organosilica nanoparticles) loaded with tumor-targeting Tumor necrosis factor-related apoptosis-inducing ligands using PMOs as carriers. *In vitro* and *in vivo* experiments have shown that the synthesized drug can still target tumors and that DOX and TRAIL can be rapidly disassembled due to the clever design of the “lysosomal shear site.” The lysosomal shear site is a clever design of DOX and TRAIL, which can be broken down rapidly. ICD marker measurements showed that the expression of CRT and HMGB1 was increased in DOX@PMO-hT-treated 4T1 cells and was significantly higher than that of free DOX, which means that ICD was enhanced. In addition, drug treatment activated CD4^+^T/CD8^+^T and guided DC cell maturation. Overall, Feng further investigated PMOs and TRAIL and found they have great potential for tumor therapy. The experimental results showed that the drug could lead to ER and oxidative stress, triggering ICD and antitumor response. The excitation efficiency of ICD was higher than free DOX’s, and DC cells were more mature. *In vivo*, experiments showed significant inhibition of both *in situ* and liver metastatic tumors after drug treatment. In summary, this experiment induced antitumor immunity in the form of laterally triggered ICD and also improved the effect of conventional ICD drugs by the application of mesoporous nanocarriers, which led to more substantial immunogenic death of tumor cells, providing a direction for future-wide application-based on mesoporous nanomaterials.

Nanomaterials with CDT properties are catalytically active in the unique environment of TME and unlike PDT and RT, he does not require external excitation such as light or radiation. Meanwhile, through precise dose control and design of nanomaterials, CDT can reduce the toxic effects on normal cells and improve the safety of treatment. Most importantly, the oxidative stress generated by CDT nanomaterials can induce tumor cell death and may release TAAs, which activate the immune system to further attack the tumor.

### 5.2 Nanomaterials involved in PDT

PDT is a low-invasive, low-toxicity treatment that damages tumor cells by cytotoxic effects through the reaction of photosensitizers with laser light to excite oxygen molecules to generate singlet oxygen. Because of its high uptake signal and sensitivity in the tumor lesion area, PDT has been widely used for imaging-guided tumor precision therapy (150–152).

**Figure 4:**
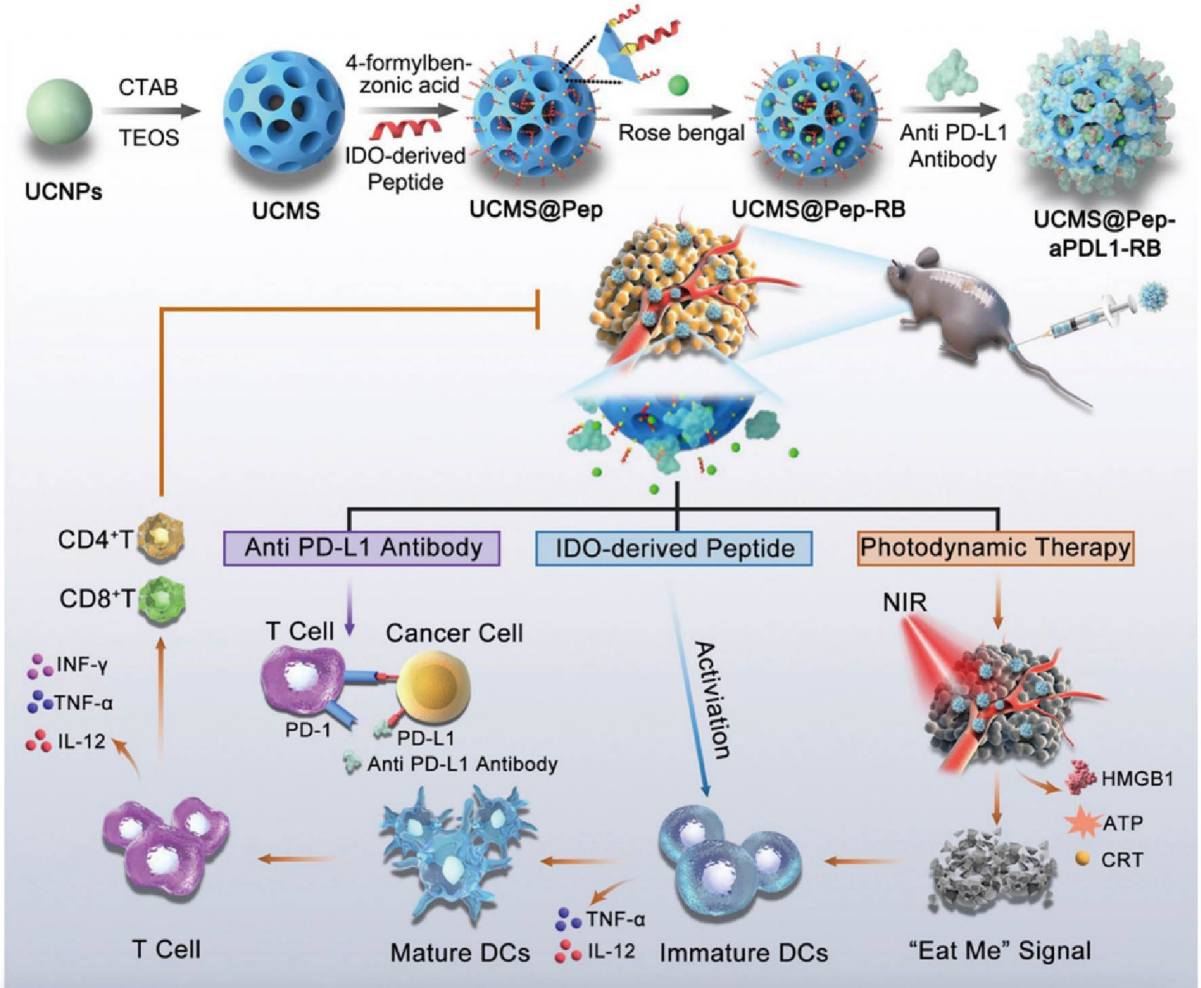
UCMS@Pep-αPDL1 induces tumor ICD and collaborates with PD-L1 to achieve tumor therapy (153). Copyright^©^ 2021, copyright Wang et al.

In recent years, ICB has gradually become the first approach for cancer treatment. However, the PD-L1 pathway suffers from a low infiltration rate of CTLs in lung cancer treatment. Research teams favor mesoporous nanomaterials with large pore sizes and high specific surface area. Inspired by the above, Dong’s team (153) combined mesoporous silica with upconversion nanoparticles to obtain a mesoporous nanocarrier UCMS, a structure with large pores to load more cargoes, such as metals, antigens, and peptides. With the carrier secured, the researchers assembled photosensitizer molecules and IDO-derived peptide vaccine AL-9 with PD-L1 inhibitor to construct the nano immune platform UCMS@Pep-αPDL1-RB. This platform proved effective in loading cargo and induced PDT and thus killed tumor cells under near-infrared light irradiation (**Figure 4**). In the process, the team also found that LLC cells release DAMPs, representative of HMGB1 with CRT levels several times higher than controls, representing the occurrence of ICD in tumor cells. Not coincidentally, the nanoplatform takes full advantage of the action of the peptide vaccine AL-9 to stimulate DC maturation, release cytokines, and activate T cells leading to tumor death. In an *in vivo* experiment, C57BL/6 mice treated with UCMS@Pep-αPDL1-RB+NIR showed massive apoptosis of tumor cells and normalization of other tissues and organs. The team analyzed the reasons for the surprising results. Firstly, the half-life of the nano-formulation is 0.96 H after entering the bloodstream, which fully utilizes the EPR effect and makes the drug content in the tumor site high and long. In addition, the PD-L1 molecules on the “transporter” effectively accumulate at the tumor site to produce the inhibitory effect, and the PDT and peptide vaccine can further expand the PD-L1 inhibitory effect. In this study, the researchers combined NIR light-mediated PDT, ICB, and peptide to enhance the immune response to achieve the most substantial therapeutic effect. These promising experimental data also provide a “shot in the arm” for treating metastatic spinal tumors, a boon to thousands of NSCLC patients.

IR-780 can effectively induce PDT and PTT for tumor killing, but its limited cycle time, high toxicity, and strong hydrophobicity have limited the further application of IR-780 (154–156). To find a suitable “ship” for IR-780, Lu et al. (154) focused on mesoporous structures and designed an MPDA (mesoporous polydopamine nanoparticle), which is a spherical structure with a 12 nm pore size and can be loaded with IR-780 with high efficiency (49.7 wt%) to form IR-780@MPDA. -In the characterized observation experiments, IR-780@ MPDA exhibited high solubility and stability in various solvents.IR-780@MPDA was also observed to increase in temperature with increasing laser irradiation time up to about 50°C while having excellent stability and ^1^O_2_ yield. IR-780@MPDA also triggered ICD, as evidenced by significantly higher levels of CRT, HMGB1, and ATP on the tumor cell surface. Tumor growth was significantly smaller than that of the control group during the same period, and these results occurred with only minimal damage to normal skin and without invasion of other normal tissues. As with the *in vitro* results, significant DAMPs were also detected *in vivo* and induced a more robust immune response under the wild growth of CD3^+^ T cells, TNF-α, IL-2, and INF-γ factors. In conclusion, this mesoporous nanoplatform effectively ameliorated the IR-780 defect and triggered the combined PDT/PTT effect, leading to promising therapeutic results. The multimodal treatment scheme is worth extending to other solid tumor treatments, broadly applying mesoporous nanomaterials and IR-780.

Nanomaterials with PDT properties have significant advantages:(1). Minimal invasiveness: the use of photosensitizers to generate reactive oxygen species under light irradiation at specific wavelengths, thus killing tumor cells, which is gentler and less damaging to patients compared to traditional surgery. (2). Controllability: The irradiation of light source can be controlled very precisely, including irradiation time, light intensity and irradiation area, thus realizing the fine regulation of treatment. (3) Repeatable treatment: PDT does not produce resistance to tumor tissue after treatment, so treatment can be repeated as needed. (4) Tissue repair: After PDT treatment, damaged tissues tend to be able to undergo natural repair relatively quickly, which helps to maintain the function of tissues and organs. However, their application still faces some challenges, such as the light penetration problem in deep tumors, the long-term stability of photosensitizers, and the safety of large-scale production and clinical application are still thorny issues that need to be overcome through continuous research and technological innovation.

### 5.3 Nanomaterials involved in PTT

PTT uses photothermal converters to convert light energy into heat to raise the temperature of the tumor site to produce a deadly effect. PTT has potent efficacy, common adverse effects, and high controllability, and it has a promising future in tumor treatment (157, 158).

**Figure 5:**
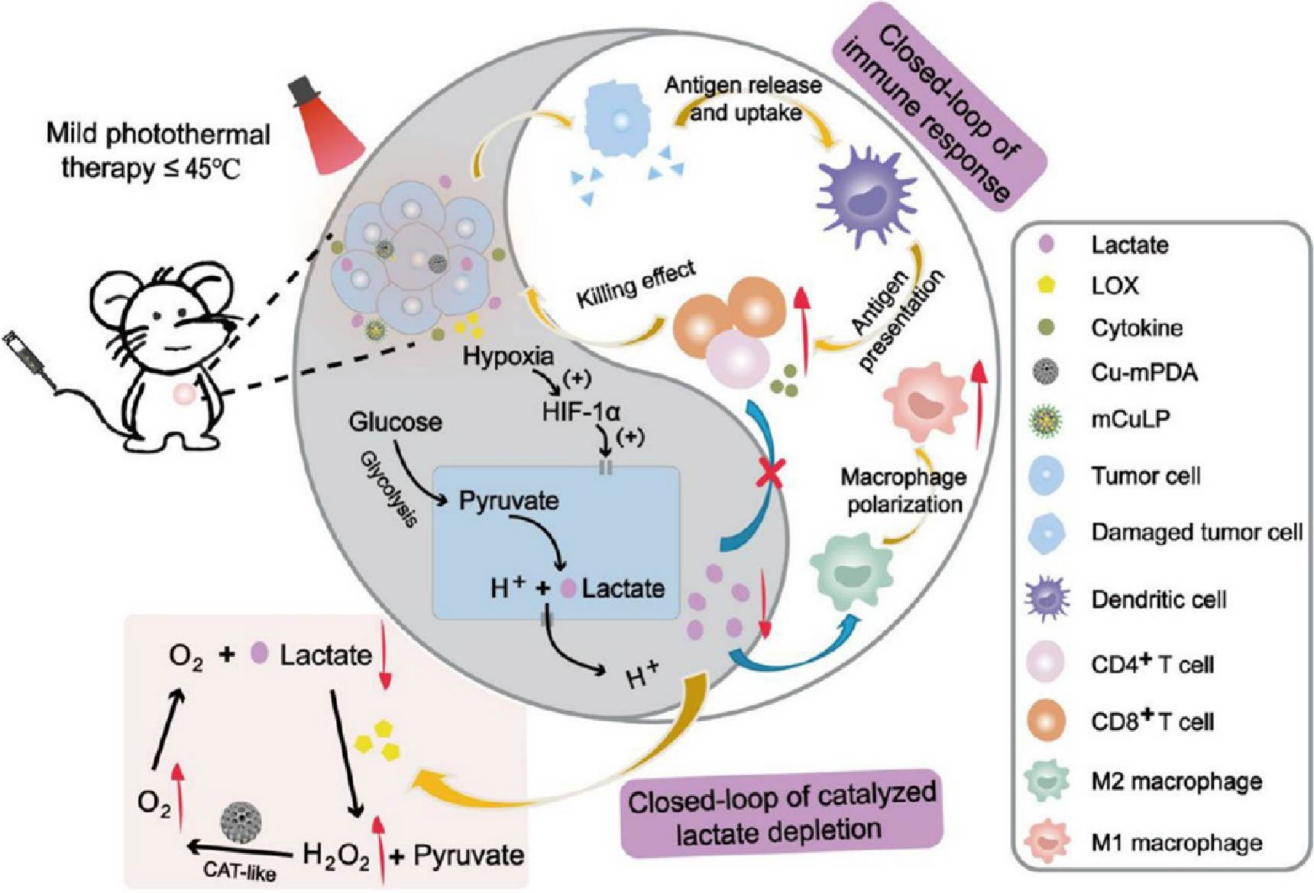
Schematic illustration of mCuLP nanomaterials enhancing photothermally induced immunotherapy by catalyzing lactic acid depletion and reshaping the TME (159). Copyright^©^ 2022, copyright Zheng et al.

The TME is often characterized by hypoxia and weak acidity due to the immunosuppressive TME brought about by lactate accumulation. Dong’s team (159) proposed the mCuLP nanosystem by integrating mesoporous polydopamine nanoparticles (mPDA) with LOX and Cu^2+^, noting that PTT can induce ICD with negligible side effects. In the characterization study, oxygen content decreased with increasing LOX concentration, and lactic acid was heavily depleted to produce H_2_O_2_. At the same time, Cu^2+^-loaded mPDA decomposed H_2_O_2_ to replenish oxygen depletion forming a cyclic system that maximized lactic acid decomposition. mPDA is a commonly used photothermal material that maintained its original conversion efficiency after loading Cu^2+^, suggesting that the mCuLP system can also trigger PTT (**Figure 5**). mCuLP showed strange effects in *in vitro* experiments, and the data showed that the nanosystem was sensitive to a high lactic acid environment, which could consume more lactic acid in the process and, more obviously, inhibit tumor cell activity. Low-temperature irradiation at 43-45°C showed a 41.5%-81.9% apoptosis rate of tumor cells, and then after a more profound analysis of the cells, it was observed that the cell surface CRT was enhanced. The nuclear HMGB1 content decreased, and there was no doubt that PTT triggered ICD. mCuLP can also increase the ratio of CD8^+^ and CD4^+^ T cells, promote the conversion of macrophages to the M1 type, induce DC maturation and T cell activation, and effectively enhance the anti-tumor immune response. This dual closed-loop strategy based on mesoporous materials effectively combines lactate depletion and immune response, which is a brilliant combination with great potential in the treatment and clinical application of solid tumors.

Silica nanoparticles are mesoporous structures with large pores, controlled drug release, and good biocompatibility, which are promising for drug transport as carriers. Based on this background, Rong et al. (160) developed a nanoplatform for AuDRM via photothermal, starvation, and immunotherapy, obtained by first loading Au NPs and R837 as coatings onto DMSNs (dendritic mesoporous silica nanoparticles) and wrapping PH-sensitive tumor cell membranes. AuDRM maintains the sensitivity of tumor cell membranes, can recognize homologous tumors, and rapidly accumulate at tumor sites. In an *in vitro* experiment simulating the TME, the weak acidic environment could make AuDRM exhibit sufficient glucose oxidase activity, appear glucose depletion, and gluconate production, which implies the detachment of PH-sensitive tumor cell membranes after entering the TME. Besides, AuDRM has excellent photothermal efficiency and stability, showing rapid warming and maintaining a constant maximum temperature under laser treatment. In *in vitro* experiments, heat shock protein 70 content was up-regulated after AuDRM and laser treatment of 4T1 cells, which represents the immunogenicity brought by PTT. Also, elevated expression of CRT, ATP, and HMGB1 confirmed the trigger of ICD, which confers immunogenicity to apoptotic tumor cells. AuDRM showed homologous targeting ability and photothermal efficiency *in vivo* experiments consistent with *in vitro* experiments and produced stronger ablation of *in situ* and metastatic tumors and prolonged survival of at least 80 days under the influence of these factors. Notably, immune memory was generated in AuDRM + Laser-treated mice, and effector memory T cells could function in a surge after reinoculation of tumors. This nanoplatform makes an excellent idea for tumor-targeted immunotherapy, not only realizing the combination of photothermal/starvation/immunotherapy but also providing a precedent for designing nanocarrier formulations for homologous tumor immunotherapy.

Mesoporous silica-coated gold NPs (Au@SiO_2_ NPs) are drug carriers with high drug loading efficiency, good photothermal performance, and modifiability. However, the drawback of uncontrollable harm to normal tissues prevents him from being used in clinical therapy. To fabricate a safe drug carrier based on Au@SiO_2_ NPs, Tao et al. (161) used PH-sensitive ZnO quantum dots to modify Au@SiO_2_ NPs to obtain AuNP@mSiO_2_@ZnO and loaded DOX to form AuNP@mSiO_2_@DOX-ZnO for therapeutic efficiency and safety evaluation. During the preparation process, the pores of Au@SiO_2_ NPs (~2.8 nm) were utterly closed by ZnO quantum dots (~5 nm), which could help to avoid drug dropout, so the system exhibited efficient drug loading. auNP@mSiO_2_-ZnO has good photothermal conversion efficiency and can rapidly increase the temperature with laser irradiation in a short period. Moreover, the ZnO “cap” can be detached rapidly in an acidic environment, allowing for efficient DOX release. In *in vitro* experiments, the carrier DOX rapidly accumulated in B16/F10 melanoma cells and TAMs within a short time, and this mechanism played a positive role in the anti-tumor response. In addition, the detached ZnO could release Zn^2+^ and thus produce cytotoxic ROS to enhance the anti-tumor effect further. 95.5% reduction in tumor volume and 96.2% reduction in weight in the subcutaneous melanoma mouse model treated with AuNP@mSiO_2_@DOX-ZnO + Laser were much higher than other groups. Not only that, but the researchers also found that ICD was triggered during the above anti-tumor process, and CRT, HMGB1, and ATP all verified this finding. ICD in tumor cells also further promoted DC cell maturation and activation of cytotoxic T cells to trigger the immune response. Encouragingly, AuNP@mSiO_2_@DOX-ZnO could also obtain similar suppressive effects as unilateral ones by triggering anti-tumor immunity in bilateral subcutaneous melanoma mouse models and lung metastasis mouse models. Notably, these therapeutic effects are based on the premise that no other normal tissues and organs are harmed in mice. This also reflects that this carrier overcomes the hazardous nature of the original Au@SiO_2_ NPs. Overall, the discovery of this new composite nanomaterial connects Au@SiO_2_ NPs carriers with solid tumors, making it possible to “talk on paper.” It also reveals the application of ZnO NPs in treating tumors, which is a feasible, safe, and efficient option for melanoma treatment.

Nanomaterials with PTT function have obvious advantages in the field of ICD-induced tumor therapy, mainly including: (1). Non-invasive or minimally invasive. (2). Efficient thermal killing: nanomaterials activated by light irradiation can quickly convert light energy into heat energy, generate local high temperatures, and effectively kill tumor cells. (3). Strong controllability: By adjusting the intensity of the light source, irradiation time, and irradiation position, the temperature of the treatment area can be accurately controlled to achieve accurate control of the treatment process. (4). Multi-mode therapy: Some PTT nanomaterials have multiple functions, such as imaging and therapeutic functions at the same time, and can be monitored in real-time while treating (162). However, the application of PTT nanomaterials also has some challenges and limitations, such as limited light penetration depth, which may be difficult to treat deep tumors; The biocompatibility and biodegradability of nanomaterials need further study. And ensuring that heat is precisely controlled to avoid thermal damage to surrounding healthy tissue. These problems need to be solved through continued scientific research and technological innovation.

Mesoporous nanomaterials hold unique advantages and disadvantages when inducing ICD in tumor cells, particularly when compared to other types of nanomaterials. One of the primary advantages of mesoporous nanomaterials is their high surface area and large pore volume, which allows for the loading and controlled release of significant amounts of drugs or bioactive molecules, enhancing therapeutic efficacy. By adjusting pore size and surface chemistry, these nanomaterials can be engineered to release drugs efficiently in specific conditions, such as the TME. The ability to functionalize mesoporous nanomaterials easily further adds to their versatility, as surfaces can be modified to attach various functional molecules like targeting agents and fluorescent markers, making them advantageous for targeted therapy, image-guided therapy, and multimodal treatments. Additionally, materials like mesoporous silica show excellent biocompatibility and biodegradability, reducing the risk of immune responses or long-term toxicity, which is often a concern with certain metal nanoparticles.

However, mesoporous nanomaterials also present several challenges, such as the complexity and cost of synthesizing highly ordered mesoporous structures. The synthesis process requires precise control of production conditions and special templating agents, complicating large-scale production consistency and practicality. Stability issues in complex physiological environments necessitate additional stabilization strategies, as nonspecific adsorption of proteins in the bloodstream can affect targeting and drug release efficiency. While mesoporous nanomaterials have a large pore volume, their drug-loading efficiency and capacity can still be limited by pore size and architecture, which may be less flexible than some polymer nanomaterials with higher loading capacities. Thus, optimizing the pore structure for larger or more complex biomolecules remains a challenge.

Furthermore, although biodegradability is an advantage, controlling the degradation rate to fit specific therapeutic needs and treatment windows can be difficult. Both overly fast and overly slow degradation rates can negatively impact therapeutic effectiveness, necessitating fine-tuning for maximizing therapeutic benefits. In summary, mesoporous nanomaterials demonstrate significant promise in inducing therapeutic ICD, offering high surface area, excellent functionalizability, biocompatibility, and tunable pore structures. Nonetheless, challenges in synthesis complexity, stability, drug-loading capacity, and biodegradation rate must be addressed through further research and optimization to improve their broad and effective application in tumor therapy.

## 6 Hydrogel nanomaterials

Hydrogel is a hydrophilic, reticulated gel with long-term stability. Hydrogels also have biocompatibility, biodegradability, and flexibility that are unmatched by other materials, making them the star material of the biological family. In recent years hydrogels are often individually designed and modified for drug delivery and have many applications in oncology therapy (163–165). Also, numerous drugs and mechanisms have been observed to significantly enhance the effects, such as ICD after gel modification. We present in this section the applications of hydrogels involved in the induction of ICD in tumors by *in situ* injection, intravenous injection, and transdermal delivery, respectively, classified by the injection mode of hydrogels.

### 6.1 *In situ* injection of hydrogel nanomaterials

*In-situ* injection is the most commonly used modality for hydrogels, and compared to other methods, drugs can directly reach the tumor site for precision medicine. ICD triggered by *in-situ* injection-based nano gels has gained extensive research and has considerable potential for clinical translation.

Considering the advantages of hydrogels, Chen et al. (166) combined phenylboronic acid-modified SN38SA-BA with PVA (polyvinyl alcohol) to construct a ROS-responsive hydrogel transport vehicle in an attempt to deliver α-PDL1. SN38 is derived from CPT (camptothecin), a product that activates the immune system and possesses anti-tumor talent. In the characterization assay, the synthesized gels were porous, and adding αPDL1 did not alter the gel properties. Besides, the hydrogel can be rapidly decomposed into SN38 and IgG in the H_2_O_2_ solution, stimulating ROS production and thus reaching the ultimate efficiency of drug release. *In vitro* experiments showed that the gel can effectively stimulate ICD, as shown by the rise in the expression of DAMPs. The degree of ICD was higher in hydrogels than in free SN38, probably due to the partial loss of the free drug. In the *in vivo* tumor model observations, PVA-SN38 exhibited the same anti-tumor immunity as *in vitro*. As expected, the “eat me,” “find me,” and “danger” signals were significantly increased, as well as the DC cell maturation rate, and these effects were superior to those of These effects were better than those of the control group and other experimental groups. Thus, the hydrogel also stimulated the tumor ICD *in vivo*, triggering a robust immune response. This experiment combined chemotherapeutic drugs with ICB and utilized the ROS-responsive catabolic mechanism, a bold innovation for SN38. This class of drugs not only achieves therapeutic effects on tumors but also enhances immunogenicity and contributes to the development of tumor immunotherapy.

IND is a potent immune checkpoint inhibitor that blocks tumor growth and metastasis by stimulating immune cells and cytokines to trigger an immune response. As research progresses, the disadvantages of poor targeting, side effects, and rapid breakdown of this mechanism gradually emerge. Tsai’s team (167) worked on nanomaterials, modified PAMAM (Poly(amidoamine)) with PCLA-PEG-PCLA triblock copolymer hydrogel to form drug carriers, and equipped with PAMAM is a homogeneous spherical nanoparticle that has been shown to exert good antitumor efficacy in combination with DOX. The hydrogel can rapidly release a considerable amount of drug in an acidic environment. The drug further makes cancer cells die, and it is noteworthy that the cell death cell effect caused by PAB-DOX/IND is comparable to free DOX and significantly more substantial than other groups. To further investigate the therapeutic effect and pro-apoptotic principle, the team injected Gel/PAB-DOX/IND near the tumors of HeLa cell transplanted mice, and the results showed that the tumor volume in this group was the smallest and very significant, and the Gel/PAB group was consistent with the control group. The level of Ki67 protein was the lowest after hydrogel treatment, and Ki67 is a marker of tumor proliferation. Further tumor section observation revealed the most significant degree of tumor necrosis in this group, and a significant increase in numerous ICD factors (IFN-β, TNF-α, and IL-6) and upregulation of CD8^+^ T cell expression were detected, together indicating that an effective tumor immune response was triggered during the treatment. This PH-responsive hydrogel successfully delivered drugs and triggered ICD and anti-tumor immunity, showing good potential for tumor suppression and will also broaden the application of IND.

Tumor immunotherapy is a groundbreaking discovery and a complete achievement. However, immunotherapy is imperfect, and various factors, such as a “cold” immune microenvironment, make it less effective than expected. Therefore, it is necessary to design a treatment that can enhance the efficacy and reduce the damage to the body. Inspired by combination therapy, Zhao et al. (168) designed and constructed MDDP, a layered hydrogel particle that transports oxaliplatin and NLG_919_ nanoparticles, which self-assembles PEG, two drugs that trigger ICD and modulate TIME, respectively, with DDP NPs (dual-drug polymeric nanoparticles) were prepared by self-assembly. Compared with free drugs, the high reducibility of DDP NPs breaks the barrier and promotes drug accumulation at the tumor site. In *in vitro* experiments, the team was surprised to find that the levels of ICD-related factors, such as CRT, ATP, HSP70, and HMGB1, were all significantly elevated in the gel-treated CT26 cancer cells, implying that the self-assembly modification did not negatively affect oxaliplatin function. Importantly, in the *in vivo* experiments, after intratumoral injection of MDDP into the tumor-bearing mice, the drug could be detected at the tumor site for a long time, and the tumor growth was slow compared with other groups. The ICD effect occurred both *in vitro* and *in vivo*; the DC cells matured extensively after MDDP treatment, and the CD3^+^CD8^+^ cytotoxic T cell numbers were also significantly hyperinflated. In addition, Treg cells were reduced approximately 2.7-fold compared to the control group, and the levels of various cytokines such as TNF-α and IFNγ were upregulated, all signs indicating that MDDP initiated an immune response. Throughout the treatment, the survival rate of mice in the MDDP group was 100%, normal tissues were not violated, and body weight was significantly increased. In conclusion, MDDP efficiently accomplished the transport function of both drugs, originally for injection to trigger ICD, change the TME, and finally effectively trigger the anti-tumor response and stop tumor growth. Therefore, we have good reasons to believe that this nano-loading approach has good prospects, and the layered hydrogel solution will undoubtedly gain more comprehensive application.

There is increasing evidence that SAzyme (single-atom nanozyme), which has peroxidase-like activity and can function as an efficient catalyst for H_2_O_2_, is an effective new cancer therapy. To overcome the limitations of the “cold” tumor environment and H_2_O_2_ deficiency for SAzyme therapy, Fan et al. designed a gel system LOA by mounting Pd-C SAzymes and CPT (camptothecin) into an agarose hydrogel was obtained. The system generates sufficient H_2_O_2_ at the tumor site and transforms the TME to treat malignant tumors with chemotherapeutic agents, ROS, and immune responses. The synthesized product undergoes photothermal conversion under light, and as the temperature rises, the gel liquefies and “explodes” to release the drug CPT. On the other hand, during the whole treatment process, all indicators of mice, such as body weight, were in normal condition, reflecting the system’s safety. DC cells matured under the influence of LOA + NIR, further enhancing CD8^+^ T levels, thus delivering a satisfactory result in inducing systemic immune responses. In conclusion, nanomaterials are a new measure to address the lack of drug permeability, enabling hyperactivity and efficient penetration into tumors through various reactions. Analogously, Liu’s team (169) constructed an AHNM (asymmetric hydrogel nanomotor) as a carrier for efficiently loaded adriamycin nano-gels AHNMs-DOX. Under laser action, gel Under the action of the laser, the gel underwent photothermal conversion to provide power to enter the tumor site. Apoptosis was detected *in vitro* and *in vivo* after injection, and the inhibition effect was evident. Notably, DOX caused an increase in the number of multiple DAMPs and TAAs to release successfully, brought about ICD, and the gel influenced these factors to exist at the tumor site for a long time, effectively promoting DC maturation and further activating the immune system. In conclusion, the active transport function of AHNMs is powerful, not only using DOX to induce ICD but also effectively preserving antigen, which provides a reference for the application of ICD in future immunotherapy.

### 6.2 Intravenous injection

Studies have shown that intravenous methods account for the most marketed nanomedicines. Intravenous injection is the most commonly used way to deliver chemotherapeutic drugs. It differs from *in situ* tumor injection in that the drug can circulate through the bloodstream after intravenous injection, allowing the blood concentration to be predicted and precisely controlled. Also, there are a variety of hydrogel products that use intravenous injections (170).

While most drugs enter the body for action by *in situ* injection, a significant number of gel products can act more efficiently through blood circulation. Cancer cells are characterized by abundant glucose metabolism, and antitumor therapy through inhibition of glucose metabolism is feasible. Mannose has been shown to interfere with glycolysis, the tricarboxylic acid cycle, and polysaccharide synthesis pathways to control cancer cell growth. Xu et al. (171) identified this mechanism, investigated it in depth by a bioresponsive crosslinking agent with a disulfide bond, and synthesized two nitrophenyl groups. Notably, the team found that this approach formed mannose-DOX gels, and multiple drugs could form stable gels with DOX. DM NGs were more readily recognized and bound by tumor cells than DOX and released by GSH depletion. DM NGs transformed the immunosuppressive microenvironment by downregulating PD-1^+^TIM-3^+^CD8^+^ T cells and induced a robust immune response by promoting DC activation through the induction of ICD. Notably, the DM NGs demonstrated their safety during the formal treatment, and the normal tissues of mice were negligibly affected. In conclusion, this DOX-based gel product has played a positive role in tumor treatment. With this research base, we can focus on other drugs and make nanogel drugs that can solve more challenges in the medical field by the DBHD approach.

siRNA is a representative transport system that can deliver numerous small-molecule drugs. To overcome this challenge, Zhang’s team (172) designed and synthesized a nucleic acid-based gel system that combines and modifies PPAs with DNA strands into a well-water-soluble PPA-DNA compound and self-assembles into a DNA framework. In *in vitro* experiments, a significant time-dependent signal was detected in treated cancer cells. Still, it gradually decreased after reaching the peak, indicating that the nanogel acts and then disintegrates. Under a 660 nm laser, the gel activates photothermal conversion to generate a large amount of ROS, leading to tumor cell death. However, these ROS do not damage the structure and function of siRNA. In addition, the disassembly process also significantly inhibited PD-L1 expression in cancer cells and suppressed tumor proliferation. In experiments on B16-F10 tumor-bearing mice, it was found that siRNA/PPA-NG released a large amount of TAAs and promoted DC maturation, further increasing the number of CTL cells, which may be a synergistic effect of ICD and ICD. In summary, siRNA/PPA-NG combined with photosensitizer synergistically with anti-PD-L1 exerted potent anti-cancer effects and showed good biosafety and photodynamic effects, providing new insights into multi-drug combination anti-cancer therapies.

Platinum drugs such as oxaliplatin have gained widespread use in treating tumors and can trigger an ICD to initiate an immune response. However, drug resistance and side effects have become an urgent issue with prolonged drug use. Some teams have demonstrated that nanocarriers encapsulating oxaliplatin or its derivatives are an excellent approach combined with NP and colloids to develop delivery systems. Inspired by the above, Benoit’s team (173) combined hyaluronic acid and polyarginine by electrostatic interaction and combined with DACHPt to constitute a new gel system DACHPt loaded NP. Reassuringly, ICD production by the gel loaded with DACHPt was unaffected, and the degree of signalings such as HMGB1 and ATP were qualified. After intravenous injection of DACHPt-loaded NP, 5-fold higher drug concentrations than free oxaliplatin were detectable in mice, representing a prolonged blood circulation of the drug by the micelles. The highlight of the experiment is the high exposure to the drug and the rapid breakdown to prevent the prolonged and random distribution of the drug in the body. This work presents a stable degradable nanosystem with uncommon functionality. Unfortunately, the study did not perform animal tumor growth assays, and it is believed that after further experiments, this platinum derivative-based therapy will gain more acceptance and be further applied in clinical treatment.

### 6.3 Transdermal drug delivery

In recent years, transdermal drug delivery techniques have been gaining popularity because of their convenience and non-invasive nature for patients unsuitable for injectable therapy or oral drug delivery. The principle of transdermal drug delivery is that the drug penetrates the skin and enters the target location. The small size of nanomaterials shows strong adaptability at this moment.

Surgical resection is the traditional treatment for malignant tumors, but postoperative complications affect the patient’s prognosis and quality of life. Gold nano is used as a photothermal agent for tumor PTT treatment, and silver nano is well established as an antibacterial agent, and combining the two may bring miraculous results. Iota carrageenan (CA) has been shown to have advantages in drug delivery and wound healing. Based on the above background, Zhao’s team (174) used CA-carrying gold silver NPs combined with poloxamer 407 (F127), PVP, and BA to construct CA-AuAgNPs-Gel with a full photothermal effect. The gel product rapidly warmed up within 6 minutes under the action of the laser, causing a strong PTT effect, which killed more than 90% of 4T1 and B16F10 cells *in vitro* and effectively suppressed tumor growth and residual tumor recurrence in tumor-bearing mice. A significant elevation of ROS was detected during treatment, and there was an increase in CRT exposure, HMGB1 release, and ATP, implying the emergence of an anti-tumor immune response. Importantly, this strategy stops bleeding by inducing erythrocyte and platelet accumulation, and it was observed that the gel-treated group could completely stop bleeding within the 30s and also lost significantly less blood than the control group. Notably, the gel also inhibited the growth of several bacteria and prevented postoperative infection. In conclusion, CA-AuAgNPs-Gel is a multifunctional nanogel with both delivery and tissue regeneration, and its discovery suggests that we do not rely solely on antibiotics for antibacterial activity. Still, hydrogel-based nanoproducts may perform well beyond traditional drugs with their personalized modifying effects.

**Figure 6:**
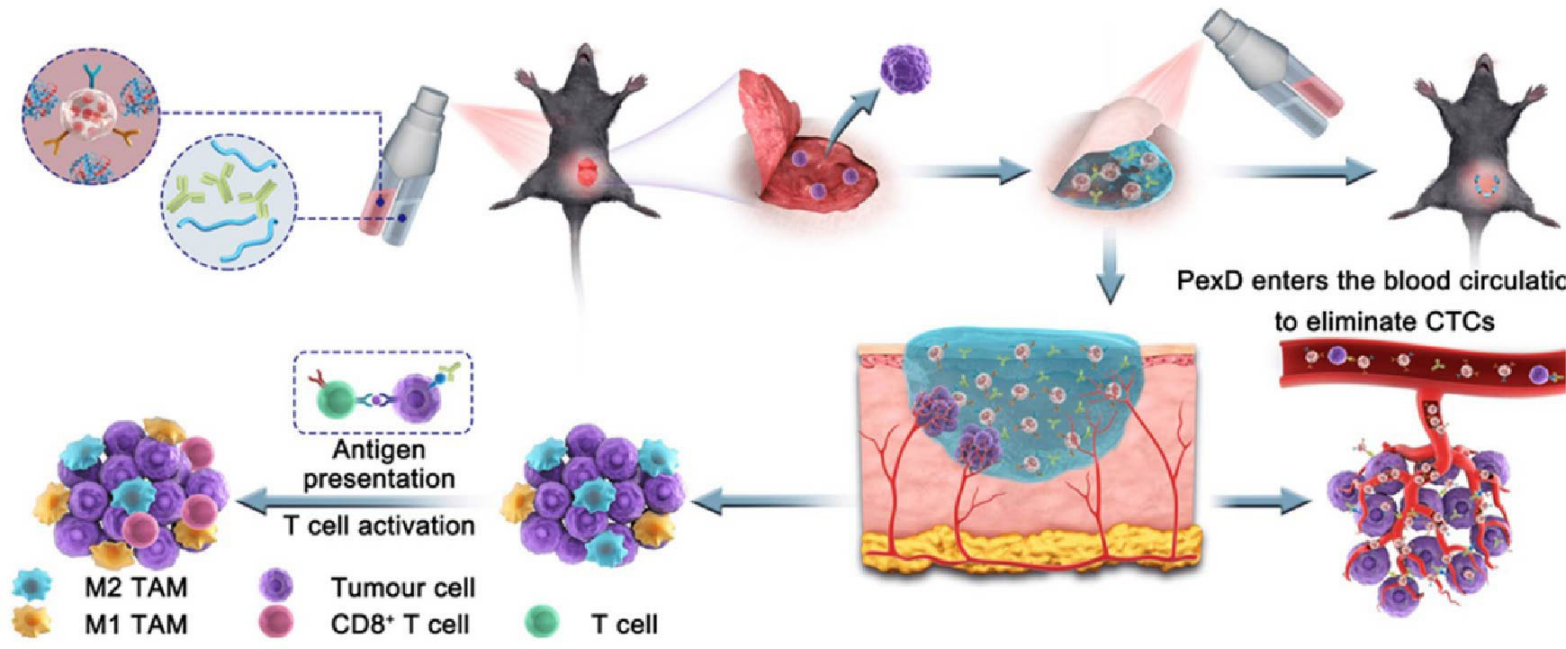
A model diagram of a spray chemical immunotherapy gel that inhibits postoperative tumor recurrence by releasing PD-L1 antibodies and platelet-derived small EVs (175). Copyright^©^ 2021, copyright Sharma et al.

EVs (Extracellular Vesicles) are nanoparticles widely distributed in various fluids *in vivo* and play essential roles in many physiological processes. Sun et al. (175) conducted preliminary experiments to demonstrate that platelet-derived small EVs (Pex) also can bind to CTCs. On this basis, the team designed a fibrin gel spray αPD-L1-PexD-Gel for therapeutic use, which was made by combining Pex with DOX and loading it into a thrombin solution with αPD-L1, resulting in a product with both PexD and αPD-L1 effects. Compared to free Pex, PexD was superior in adhering to tumors, probably due to the high affinity of its surface P-selectin for CD44 receptors on the tumor surface (**Figure 6**). DOX itself can trigger ICD, but *in vitro*, experiments found that PexD-treated cancer cells exhibited significantly higher CRT expression and significantly higher levels of ATP and HMGB1 than free DOX, which represents. In addition to the significant inhibitory effect on mouse metastases and consistent with *in vitro* experiments, the team also found that αPD-L1-PexD-Gel sprayed on mouse tumor sites had a significant advantage over other groups in suppressing Treg cells and upregulating CD8^+^ T cells, which is a strong argument for initiating T cell immune response. Also, the spray promoted TAMs to M1 sexual polarization, which further played a preventive and therapeutic role against tumor metastasis and recurrence. Overall, the study focused on the postoperative period of melanoma and cleared metastases by the inhibitory effect of ICD with αPD-L1. Moreover, this spray is simple to prepare and handle and is a promising tool for a new clinical treatment strategy.

The transdermal drug delivery technique overcomes the difficulties of random distribution of intravenous drugs and uneven distribution of *in situ* injections, achieves drug infiltration into tumor skin lesions, and has the advantages of simple preparation, safety, flexibility, and painlessness for patients. Gao et al. (176) noticed this technique. They studied and synthesized a nano hydrogel microneedle L-Ce6 MNs acting on the skin surface. The components were formed by self-assembly of PUFAs and Ce6 to form L-Ce6 NAs, which were later loaded onto the tip of MNs (microneedles) to constitute. It is gratifying that MNs efficiently delivered the front-end drug to a depth of about 600 μm under the skin of mice. Tumor cells showed superb uptake of L-Ce6 NAs and drug-induced ICD and ROS production under light conditions, mechanisms that are positive for tumor treatment. *In vivo* experiments also demonstrated the same effects as *in vitro*. After three treatments of L-Ce6 MNs once every three days for a total of three times, microneedle triggered ICD and DC maturation bringing about T-cell immune responses compared to all other groups, which showed direct tumor growth inhibition and significantly prolonged survival. Notably, mice with metastatic tumors also died extensively, and the skin punctured by microneedles could be fully recovered within 18 days. Of course, to avoid damaging the normal skin, it is crucial to cover the normal area during treatment and to try to administer the drug only within the tumor area. Similarly, Zhou’s team (142) constructed a gel microneedle PDM by combining PST, β-catenin silencing DNAzyme, and MOF containing TA and Mn^2+^. In *in vivo* experiments, the microneedle was applied to the tumor site and penetrated the tumor through the skin, combining PTT with DZ. In *in vivo* experiments, the microneedle was applied to the tumor site and penetrated the tumor through the skin. Of course, during this process, PTT induced ICD by releasing various DAMPs, further enhancing the anti-tumor effect.

Hydrogel-based nanomaterials represent a promising avenue in cancer therapy, particularly for inducing ICD in tumor cells when combined with other therapeutic modalities. Their unique physicochemical properties and tunable functionalities enable them to serve as effective delivery platforms and therapeutic agents. The inherent biocompatibility and biodegradability of hydrogels make them suitable for clinical applications, capable of encapsulating a wide range of therapeutic agents such as small-molecule drugs, proteins, peptides, and nucleic acids. This encapsulation protects these cargoes from rapid degradation and enhances their stability in biological environments, making controlled and sustained release of immunogenic agents possible. This characteristic is especially advantageous for triggering ICD, as it enhances the activation of the immune response against tumor cells.

Moreover, hydrogels can be engineered to respond to various stimuli such as pH, temperature, enzymes, or light, enabling precise control of drug release in the tumor microenvironment. This stimuli-responsive behavior can synchronize the release of ICD-inducing agents with other treatments like chemotherapy, photodynamic therapy, or radiation therapy, thereby significantly enhancing overall therapeutic efficacy. For instance, a pH-responsive hydrogel could release chemotherapeutic drugs specifically in the acidic tumor microenvironment while simultaneously delivering ICD inducers to bolster the immune response. The structural versatility of hydrogels also allows for the incorporation of various functional elements, such as nanoparticles, therapeutic antibodies, or cytokines within a single delivery system. This multifunctional capability facilitates the design of potent combination therapies targeting different pathways involved in tumor growth and immune evasion, thus providing a synergistic therapeutic effect. Furthermore, the ability of hydrogel-based nanomaterials to offer localized delivery minimizes systemic toxicity and enhances the concentration of therapeutic agents at the tumor site, maximizing therapeutic impact while reducing adverse effects on healthy tissues.

However, several challenges remain in the application of hydrogel-based nanomaterials for ICD induction in combination therapies. The complexity of designing multifunctional hydrogels that can appropriately release multiple therapeutic agents in a controlled manner requires significant optimization. Ensuring the stability and scalability of these hydrogel systems for clinical use is also a critical concern. Additionally, understanding the interactions between hydrogel components, the encapsulated agents, and the biological milieu is crucial for predicting and controlling therapeutic outcomes.

In summary, hydrogel-based nanomaterials exhibit significant potential in inducing ICD when used in concert with other therapeutic modalities. Their biocompatibility, stimuli-responsiveness, structural versatility, and ability to provide localized delivery make them ideal candidates for enhancing the efficacy of combination cancer therapies. Nonetheless, addressing the challenges in design, stability, and scalability is essential for translating these promising systems into clinical applications. Through continued research and development, hydrogel-based nanomaterials could become a cornerstone in the next generation of cancer immunotherapies, amplifying the effectiveness of combined therapeutic strategies.

## 7 Conclusions and perspectives

In this review article, we provide an in-depth analysis of the advances in the application of a range of nanomaterials with cutting-edge properties in the induction of ICD. Specifically, we focus on such kinds of nanomaterials as biological cell membrane modification techniques, self-assembled nanostructures, metallic nanoparticles, mesoporous materials, and hydrogels. These materials show great potential in the field of tumor immunotherapy due to their unique physicochemical properties and functionality. Our classification approach aims to provide a clear framework for the research community to better understand the mechanism of action and application prospects of various types of nanomaterials in stimulating ICD. However, we also recognize that the functions of nanomaterials are often not limited by a single class and that they can interact in a variety of ways and play important roles in different biological processes. Undeniably, although these nanomaterials have performed well in laboratory studies, they still face some challenges in practical applications. For example, complex synthesis and modification processes may limit their large-scale production, and potential toxicity and side effects on biological systems need to be evaluated and optimized through further toxicological studies. In addition, the biodistribution, metabolism, and excretion mechanisms of nanomaterials need to be thoroughly investigated to ensure their safety and efficacy. Furthermore, including case studies where nanomaterials have shown success in preclinical trials can help fortify our understanding of their clinical potential. In conclusion, with the continuous advancement of nanotechnology and biomedicine, the prospect of these nanomaterials in inducing ICD and their application in tumor immunotherapy is promising. Future research will need to focus on addressing the technical and biological barriers facing the clinical translation of these materials, as well as developing new strategies to enhance their therapeutic efficacy and provide safer and more effective therapeutic options for patients.

In this context, emerging technologies at this stage, such as molecular docking, artificial intelligence (AI), and machine learning, provide new strategies and tools for nanomaterials in inducing tumor ICD by influencing their synthesis, functionalization, and application. Molecular docking technology serves as a powerful computational simulation tool that allows us to predict and analyze the interactions between nanomaterials and key biomolecules in cells at the molecular level. In this way, we can efficiently screen and optimize the surface properties of nanomaterials to enhance their ability to induce ICD, leading to more precise drug delivery system design. Meanwhile, with the growing use of AI and machine learning technologies in materials science, their capabilities in data processing and pattern recognition provide unprecedented speed and precision in the discovery and optimization of nanomaterials. AI algorithms can extract valuable information from vast amounts of chemical and biological data to predict the biocompatibility and therapeutic efficacy of nanomaterials, thus assisting researchers at an early stage to exclude candidate materials that may be toxic or inefficient at an early stage. These emerging technologies not only provide powerful support for the synthesis and characterization of nanomaterials but also greatly facilitate the application of nanomaterials in tumor immunotherapy. By fine-tuning the physicochemical properties of nanomaterials, we can promote their effective aggregation in the TME and stimulate a strong ICD response, thereby enhancing the effectiveness of tumor immunotherapy. Additionally, conducting comparative analyses with existing cancer treatments can underscore the unique benefits offered by nanomaterials in inducing ICD. Unlike apoptotic cell death, which is typically immunologically silent or even tolerogenic, ICD has the distinct advantage of transforming dying tumor cells into a source of antigens that stimulate a robust antitumor immune response. Additionally, other forms of regulated cell death, such as pyroptosis and ferroptosis, also have immunogenic potential, but ICD is unique in its ability to simultaneously activate both innate and adaptive immune responses through the release of DAMPs and exposure of CRT. This multifaceted immunogenic response makes ICD an especially valuable approach in overcoming tumor-associated immune evasion mechanisms and enhancing the efficacy of cancer immunotherapy. It is also important to note that ICD and other forms of cell death (such as apoptosis, pyroptosis, and ferroptosis) are not absolutely independent; they may mutually influence each other. For example, certain intracellular stress responses or signaling pathways might simultaneously trigger both apoptosis and elements of ICD, thus creating a complex landscape of cell death that can modulate immune responses. Understanding these interactions can help in designing more effective combination therapies that leverage multiple cell death pathways for enhanced immunogenicity. With the continuous development and refinement of these technologies, we have reason to believe that nanomaterial-induced ICD will become an important tool in tumor therapy in the future, bringing more personalized and efficient treatment options to patients.

With the incorporation of these advanced technologies, many ICD nanoinducers in the preliminary research stage are expected to leap to clinical applications faster. Ethical considerations, including long-term safety studies, will also be crucial as we move towards clinical implementation. We expect that shortly, with the help of these high technologies, more efficient and safe nanoparticles can be developed, so that they can be transformed into practical means of clinical treatment, bringing hope to patients and safeguarding human health.
